# Recent trends and advances in chloroplast engineering and transformation methods

**DOI:** 10.3389/fpls.2025.1526578

**Published:** 2025-04-17

**Authors:** Muralikrishna Narra, Issei Nakazato, Brittany Polley, Shin-ichi Arimura, Grant N. Woronuk, Pankaj K. Bhowmik

**Affiliations:** ^1^ Aquatic and Crop Resource Development, National Research Council of Canada (NRC), Saskatoon, SK, Canada; ^2^ Graduate School of Agriculture and Life Sciences, The University of Tokyo, Tokyo, Japan; ^3^ Relica Genomics, Saskatoon, SK, Canada

**Keywords:** chloroplast transformation, SWNTs, CRISPR, nanotechnology, RNAi, biolistics, synthetic biology, metabolic engineering

## Abstract

Chloroplast transformation technology has become a powerful platform for generating plants that express foreign proteins of pharmaceutical and agricultural importance at high levels. Chloroplasts are often chosen as attractive targets for the introduction of new agronomic traits because they have their own genome and protein synthesis machinery. Certain valuable traits have been genetically engineered into plastid genomes to improve crop yield, nutritional quality, resistance to abiotic and biotic stresses, and the production of industrial enzymes and therapeutic proteins. Synthetic biology approaches aim to play an important role in expressing multiple genes through plastid engineering, without the risk of pleiotropic effects in transplastomic plants. Despite many promising laboratory-level successes, no transplastomic crop has been commercialized to date. This technology is mostly confined to model species in academic laboratories and needs to be expanded to other agronomically important crop species to capitalize on its significant commercial potential. However, in recent years, some transplastomic lines are progressing in field trials, offering hope that they will pass regulatory approval and enter the marketplace. This review provides a comprehensive summary of new and emerging technologies employed for plastid transformation and discusses key synthetic biology elements that are necessary for the construction of modern transformation vectors. It also focuses on various novel insights and challenges to overcome in chloroplast transformation.

## 1 Introduction

In plants, DNA is located in three distinct compartments: the nucleus, chloroplasts, and mitochondria, each of which carries its own genome and expresses heritable traits (Sugiura, 1992). Chloroplasts are endosymbiotically derived from once free-living bacteria and possess largely prokaryotic gene expression machinery and regulatory mechanisms (Barkan and Goldschmidt-Clermont, 2000). Owing to their origins as independent photosynthetic microbes, plastids serve as biosynthetic centers that carry out important metabolic activities including photosynthesis, sequestration of carbon, production of starch, and synthesis of amino acids, fatty acids, and pigments. They are also key compartments for sulfur and nitrogen metabolic processes (Kang et al., 2003). Chloroplasts have become attractive alternative targets for genetic engineering approaches, largely because of their capacity for overexpressing foreign genes and their maternal mode of inheritance (Gosal et al., 2009). Furthermore, chloroplast engineering technology has advantages over nuclear transformation techniques, as it enables the precise targeting of expression cassettes within the chloroplast genome (Daniell et al., 2016). It also enables polycistronic expression and eliminates the position effects by integrating transgenes through homologous recombination (Bock, 2007). The ability to produce stable chloroplast transformants has progressed considerably, from initial successes in tobacco (*Nicotiana tabacum* L.) to more than 20 species of flowering plants, including both dicots and monocots (Svab et al., 1990) (**Table 1**). These advances have opened up exciting possibilities for introducing novel genes of pharmaceutical (Davoodi-Semiromi et al., 2010), industrial (Verma et al., 2010), and agronomic (De Cosa et al., 2001) importance into crop plants. Despite these successes, there are still challenges that must be overcome to expand the usefulness of this powerful technology. Limited protocols for efficient plant regeneration and antibiotic selection in many crop species remain significant obstacles to the expansion of reliable chloroplast transformation systems across the plant kingdom (Bock, 2007). This review discusses novel approaches developed in chloroplast genetic transformation in recent years and highlights advancements in the understanding of the synthetic elements required for designing and constructing transformation vectors for multiple gene expression. New insights and challenges associated with chloroplast engineering and transformation are also summarized.

**Table 1:** Plastid transformation methods and selection conditions developed for different plant species.

Plant species	Method	Selection	Genes expressed	References*
*Nicotiana tabacum*	Biolistics	Spec^R^	*rrn16*	Svab et al., 1990
*Nicotiana tabacum*	PEG	Spec^R^	*rrn16*	Golds et al., 1993
*Nicotiana tabacum*	Biolistics	Kan^R^	*npt*II	Carrer et al., 1993
*Nicotiana tabacum*	Biolistics	Spec^R^	*uidA*	Staub and Maliga, 1995
*Arabidopsis thaliana*	Biolistics	Spec^R^	*aadA*	Sikdar et al., 1998
*Solanum tuberosum*	Biolistics	Spec^R^	*aadA & gfp*	Sidorov et al., 1999
*Nicotiana tabacum*	Biolistics	Spec^R^	*hST*	Staub et al., 2000
*Nicotiana tabacum*	Biolistics	Spec^R^	*Bt*	De Cosa et al., 2001
*Nicotiana tabacum*	Biolistics	Spec^R^	*bar & aadA*	Lutz et al., 2001
*Solanum lycopersicum*	Biolistics	Spec^R^	*aadA*	Ruf et al., 2001
*Brassica napus*	Biolistics	Spec^R^	*aadA & cry1Aa1*	Hou et al., 2003
*Lesquerella fendleri*	Biolistics	Spec^R^	*aadA & gfp*	Skarjinskaia et al., 2003
*Daucus carota*	Biolistics	Spec^R^	*Badh*	Kumar et al., 2004a
*Gossypium* spp.	Biolistics	Kan^R^	*aphA-6*	Kumar et al., 2004
*Glycine max*	Biolistics	Spec^R^	*aadA*	Dufourmantel et al., 2004
*Petunia hybrida*	Biolistics	Spec^R^&Strep^R^	*aadA& gusA*	Zubkot et al., 2004
*Lactuca sativa*	PEG	Spec^R^	*gfp*	Lelivelt et al., 2005
*Lactuca sativa*	Biolistics	Spec^R^	*gfp*	Kanamoto et al., 2006
*Brassica oleracea*	PEG	Spec^R^	*aadA*	Nugent et al., 2006
*Populus alba*	Biolistics	Spec^R^	*gfp*	Okumura et al., 2006
*Oryza sativa*	Biolistics	Spec^R^	*aadA & gfp*	Lee et al., 2006
*Brassica capitate*	Biolistics	Spec^R^&Strep^R^	*aadA & uidA*	Liu et al., 2007
*Beta vulgaris*	Biolistics	Spec^R^	*aadA & gfp*	De Marchis et al., 2009
*Solanum melongena*	Biolistics	Spec^R^	*aadA*	Singh et al., 2010
*Brassica napus*	Biolistics	Spec^R^	*aadA*	Cheng et al., 2010
*Nicotiana tabacum*	Biolistics	Gaba^R^	*GSA, hemL & aadA*	Bellucci et al., 2015
*Nicotiana tabacum*	Biolistics	Spec^R^	*gfp &bFGF*	Wang et al., 2015
*Nicotiana tabacum*	Biolistics	Spec^R^	*aadA & ArDH*	Khan et al., 2015
*Scoparia dulcis*	Biolistics	Spec^R^	*aadA & gfp*	Muralikrishna et al., 2016
*Nicotiana tabacum*	Biolistics	Tobra^R^	*aac6-aph2*	Tabatabaei et al., 2017
*Momordica charantia*	Biolistics	Spec^R^	*aadA*	Narra et al., 2018
*Capsicum annuum*	Biolistics	Spec^R^	*aadA*	Kota et al., 2019
*Saccharum officinarum*	Biolistics	Spec^R^	*aadA & gfp*	Mustafa and Khan, 2020
*Artemisia annua*	Biolistics	Spec^R^	*aadA & gfp*	Kaushal et al., 2020
*Nicotiana tabacum*	Biolistics	Spec^R^	*aadA, EGF*& *gfp*	Wang et al., 2023
*Nicotiana tabacum*	Biolistics	Spec^R^	*TrAA9B*, *TaAA9B & aadA*	Tamburino et al., 2023
*Actinidia chinensis*	Biolistics	Spec^R^	*aadA & gfp*	Chen et al., 2024

PEG, polyethylene glycol; Spec^R^, spectinomycin; Strep^R^, streptomycin; Gaba^R^, gabaculine; Tobra^R^, tobramycin.

*Indicated only selected reports year-wise up to 2024 due to length constraints.

## 2 Plastid transformation technology

There are two key aspects necessary for chloroplast genome engineering: the construction of transformation vectors, which typically include flanking sequences for homologous recombination and regulatory elements such as promoters, 5’ untranslated regions (UTRs), 3’ UTRs, terminators, intercistronic expression elements (IEEs), and cognate binding sites in plastid vectors for pentatricopeptide repeat (PPR) RNA-binding proteins, all of which regulate the expression of gene cassettes; and gene delivery methods for inserting exogenous genes along with selectable markers into the native chloroplast genome.

### 2.1 Construction of chloroplast transformation vectors

#### 2.1.1 Flanking sequences

**Figure 1:**
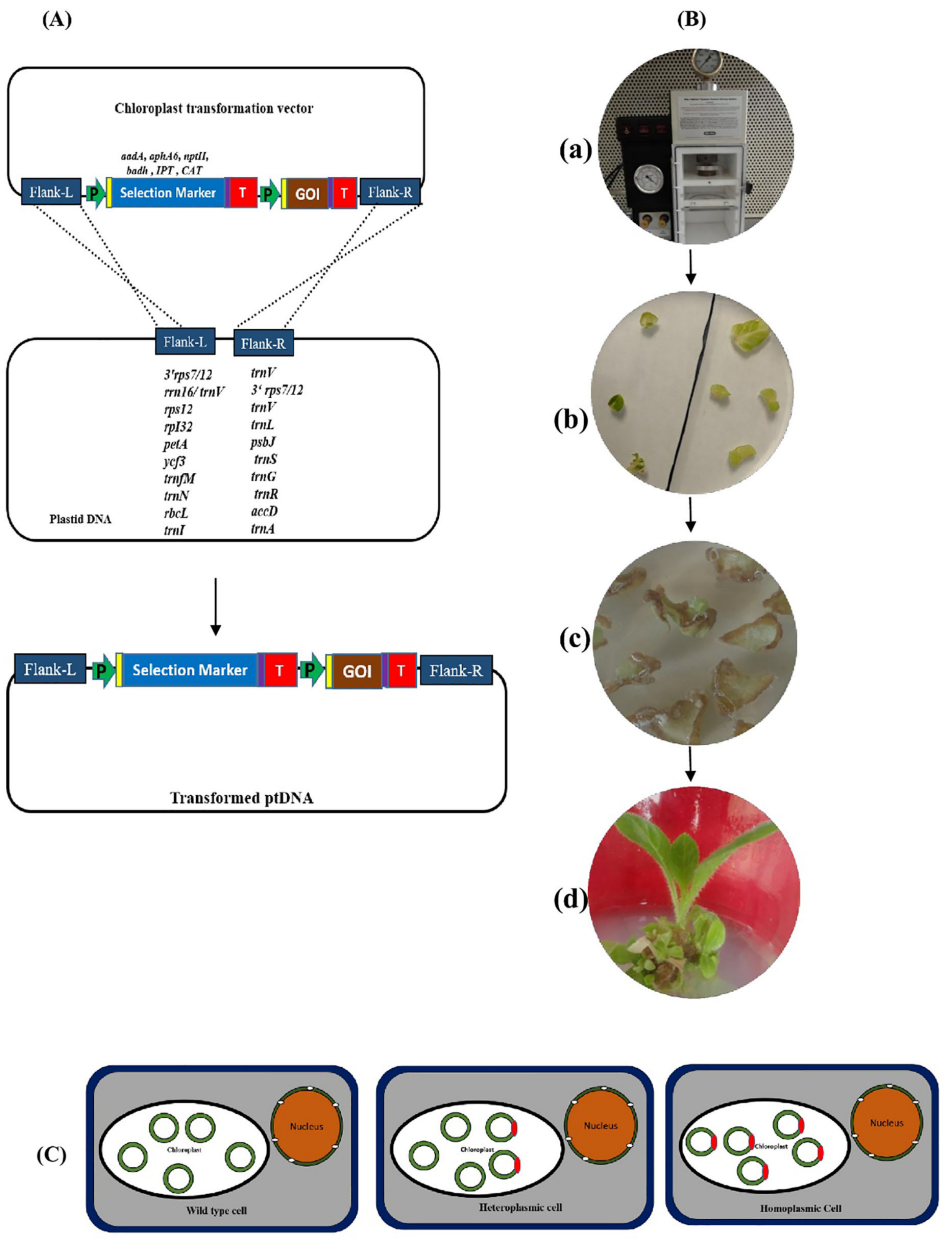
Schematic representation of the plastid transformation method. **(A)** Process of homologous recombination for integration of the gene of interest (GOI) and selection marker (SM) cassette into the native plastid genome. A typical cassette comprises a promoter (P), 5’ UTR (yellow boxes), coding region, 3’ UTR (blue boxes), and terminator (T). **(B)** Gene delivery into chloroplasts in leaf explants (tobacco as an example; *Source: unpublished photographs of Muralikrishna Narra*) using a biolistic delivery system (a) The gold or tungsten microparticles coated with desired plasmid DNA were shot on to the abaxial surface of explants using a gene gun. (b) The bombarded leaves were cut into small discs and placed on regeneration media with the appropriate concentration of antibiotics. (c) The explants were subjected to two or three rounds of selection to obtain homoplasmic shoots within 2–3 months. (d) The regenerated transplastomic plantlet. **(C)** Different stages of plant cells that have undergone plastid transformation (Wild-type cell with native plastid genome, heteroplasmic cell with a mixture of both transformed and untransformed copies of plastid genomes, and homoplasmic cell with fully transformed plastid genomes).

The insertion of foreign gene sequences into plastid DNA is typically carried out at the genetic level via homologous recombination, where the expression cassette is flanked by DNA sequences (usually between 1 and 1.5 kb in size) that are homologous to the native chloroplast genome (Iamtham and Day, 2000; Klaus et al., 2004). Many intergenic sites for transgene incorporation have been explored, but limited research has been done to examine the expression of similar transgenes at different integration sites (Maliga, 2004). The selection of target loci in the plastome may have an effect on the level of protein accumulation (Bock, 2014). The *trnI-trnA* (Daniell et al., 1998) and *trnV-3’rps12* (Skarjinskaia et al., 2003) sites, located in the inverted repeat (IR) region of the plastome, allow rapid gene copying into IR_A_ and IR_B_ regions. Integration of the transgene into these transcriptionally active spacer regions was found to be effective due to gene dosage, which resulted in increased expression levels in chloroplasts (Herz et al., 2005). The *rbcL-accD* (Svab and Maliga, 1993) and *trnfM-trnG* (Ruf et al., 2001) sites are other commonly used locations, situated in the large single copy region (LSC) of the plastome, and a gene inserted into these regions should have only one copy present per ptDNA (plastid DNA) (**Figure 1A**). Details of chloroplast vector systems mentioned in previously published research articles provide additional information regarding the factors to consider while choosing and designing a vector; interested readers can find more details in those articles (**Table 1**).

#### 2.1.2 Selectable marker genes

Selectable marker genes are crucial for the identification and positive selection of transformants, enabling the generation of plants with uniformly transformed plastids (Maliga, 2003) (**Figure 1A**). Point mutations in the 16S rRNA sequence that cause spectinomycin and streptomycin insensitivity have led to the creation of “binding-type” vectors, which contrast with more commonly used heterologous marker genes, such as aminoglycoside adenylyl transferase (*aadA*) (Kavanagh et al., 1999; Nugent et al., 2005; Svab et al., 1990). The *aadA* gene is the most efficient for chloroplast transformation due to its strong dual resistance to spectinomycin and streptomycin, making the selection relatively unambiguous across a variety of tissue types (Svab and Maliga, 1993; Ye et al., 2001). “Binding-type” markers eliminate the need for foreign antibiotic resistance genes and provide a different means of selection for studying antibiotic insensitivity. This has been successfully demonstrated in tobacco protoplast systems (Craig et al., 2008). Alternative marker genes employed for selection include *npt II* (Carrer et al., 1993), *aphA-6* (Huang et al., 2002), *cat* (Li et al., 2011), and tobramycin (Tabatabaei et al., 2017). The integration of nuclear genetics and plastid genome manipulation has enabled the development of a novel selection method, which was recently applied in ACC2-knockout lines (*Acetyl-CoA carboxylase*) of *Arabidopsis* through plastid transformation. Elimination of nuclear ACC2 function simplifies the procedure for efficient selection, resulting in the generation of spectinomycin-resistant plastid transformants at an ~100-fold enhanced frequency (Yu et al., 2017). In another study, fertile transplastomic *Arabidopsis* plants were generated using root-derived microcalli via biolistic transformation at high frequency in CRISPR/Cas9-generated ACC2-knockout explants. Null mutations in *ACC2*, which encodes a plastid-targeted acetyl-coenzyme A carboxylase, cause hypersensitivity to spectinomycin, making the selection procedure more efficient (Ruf et al., 2019). Okuzaki et al. (2020) recently reported a new strategy of using the *barnase*–*barstar* system for the production of homoplasmic plastid transformants. This system uses both nuclear-transformed estradiol-inducible *barnase* with a plastid transit signal and a plastid targeting vector with *barstar*. Heteroplasmic plants that contain wild-type plastids will be damaged when treated with estradiol due to *barnase* activity, but transformed plastids expressing *barstar* will be rescued and remain active, promoting a homoplasmic condition.

#### 2.1.3 Marker excision elements

Introduced selectable marker genes may need to be eliminated from transplastomic plants in order to address biosafety, commercial applicability, and public acceptance issues (Maliga, 2003). From a productivity perspective, expression of the marker gene in transplastomic lines should impose minimal metabolic burden once homoplasmy is achieved. Several strategies have been developed to eliminate antibiotic-resistant markers in the final products (Daniell et al., 1998; Maliga, 2003). Homology-based excision is the simplest approach for excising a marker gene, relying on the plastid’s native homologous recombination machinery, which excises any sequence between two directly oriented repeats (Iamtham and Day, 2000). Excision by phage site-specific recombinases relies on the removal of a marker gene from transplastomic plants flanked by two directly oriented recombinase target sites through the introduction of a nuclear-encoded, plastid-targeted recombinase gene (Kittiwongwattana et al., 2007). Formation of a transient cointegrate structure for the marker gene, which is positioned outside the targeting region during the initial recombination with one of the targeting sequences, causes the marker gene to be subsequently lost during a second recombination event, which occurs after the removal of antibiotic selection (Klaus et al., 2004). The co-transformation segregation approach involves target insertions at two different locations in the plastid genome with two constructs: one carrying a selective marker and the other carrying a non-selected gene. The selection of transplastomic clones for the marker gene will also carry an insertion of the non-selected gene (Ye et al., 2003). Although these advancements in the development of antibiotic resistance gene (ARG)-free crops offer hope for their commercialization in the agricultural market, they still all have undesirable limitations, such as the requirement for additional culturing, low transformation frequency rates, and a lack of testing beyond model crops.

#### 2.1.4 Regulatory sequences

**Figure 2:**
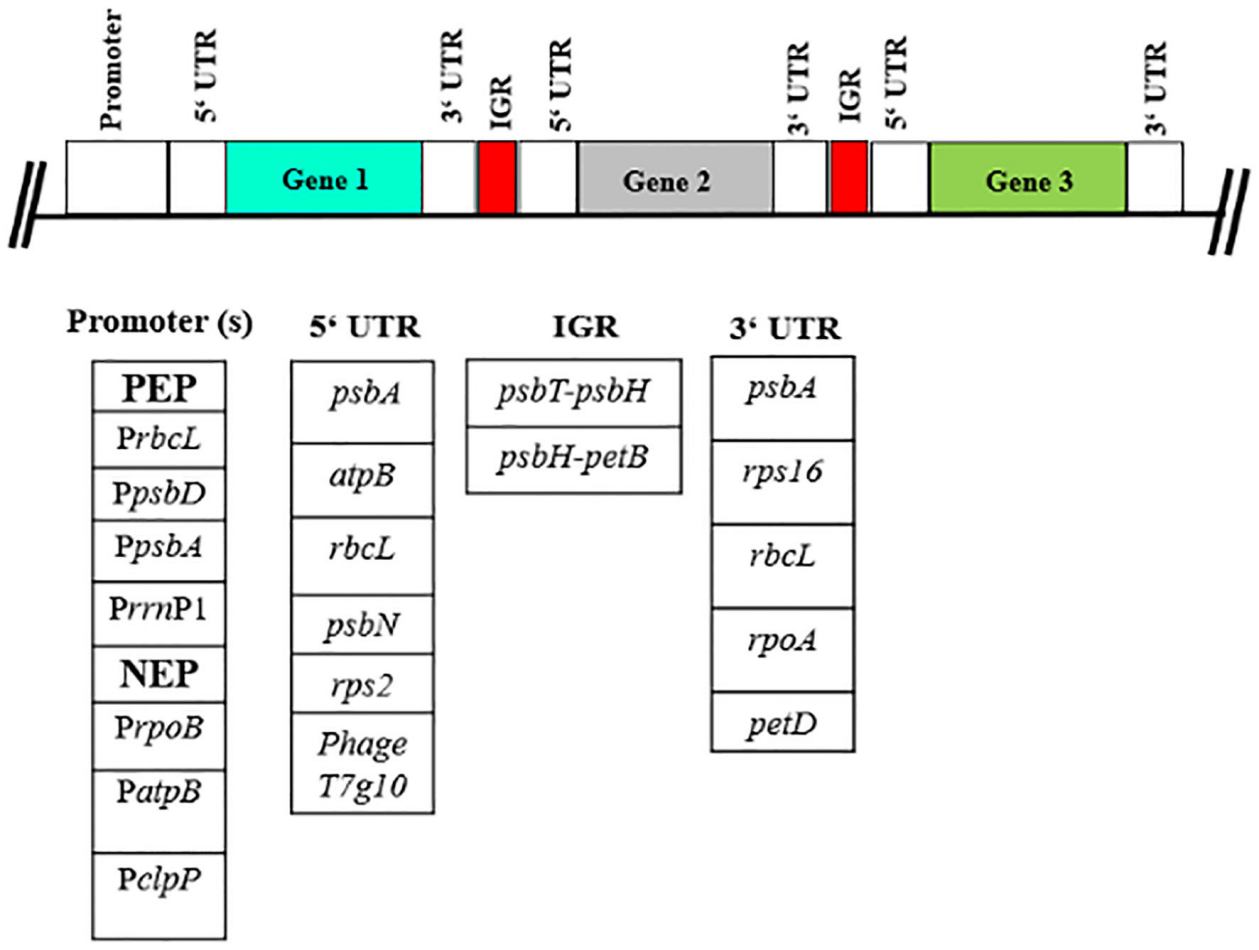
Segmental design of a synthetic plastid operon for plastid metabolic engineering. Schematic representation to show the variety of regulatory elements that can be employed for the construction of multigene operon. (IGR, intragenic plastid regions; UTR, untranslated Regions).

In chloroplast transformation, genes can be expressed in operons that encompass entire metabolic pathways, with regulation occurring at the translational level (Stern et al., 1997). Understanding the interplay between multigene operons and modified regulatory elements is essential for advancing synthetic biology-enabled plastid engineering (**Figure 2**). There are several factors that should be considered, including:

**(a) PEP promoters**

Most plastid-encoded RNA polymerase (PEP) promoters have coevolved with bacterial-type RNA (σ^70^) polymerases, which contain -35 (TTGACA) and -10 (TATAAT) consensus sequences as core motifs (Shiina et al., 2005) (**Table 2**). One of the best-studied examples of PEP promoters is one that ensures transcription of the *psbA* gene (P*psbA*), which is developmentally activated by light *in vivo.* This promoter has an additional TATA box and a prokaryotic type TGn motif in between the -35 and -10 elements. *In vitro* characterization of the mustard and barley *psbA* promoter revealed that the presence of the -35 element was essential for enhanced transcription rates (Eisermann et al., 1990). However, studies in wheat showed that removal of the −35 consensus sequence does not affect transcription in mature chloroplasts if the TGn motif is present, but rather inhibits transcription in immature chloroplasts (Satoh et al., 1999). Hayashi et al. (2003) studied alterations at the −35 consensus sequence and found that these significantly decreased the transcription rate in tobacco. Another light-activated PEP promoter, P*psbD*, has two additional regulatory elements: the AAG box and the PGT box, located upstream of the −35 consensus sequence (Thum et al., 2001). The transcription process was abolished when the AAG box was removed, even if the PGT box was retained. Likewise, alterations within the AAG box have reduced transcription by five times without affecting light inducibility. However, the removal of the PGT box did not affect the promoter’s response to light in initiating transcription (Allison and Maliga, 1995). The plastid rRNA operon promoter *PrrnP1* is another commonly used PEP promoter for transgene expression in plastids. The essential role of the -35 promoter element in *PrrnP1* was investigated through mutagenesis of the core promoter region (-37 to -8), which had a significant effect on transcription. Mutation of ACG significantly reduced the transcription rate to 8.19%, while alteration of TTG nearly abolished it (reducing transcription to 1.77%). However, mutations affecting the -10 promoter element (-16/-8 region) only moderately reduced *in vitro* transcription. Additionally, mutagenesis of the G-rich sequence (G-patch) between nucleotides -28 and -23 decreased transcription activity by 30% (Suzuki et al., 2003). With the application of improved bioinformatics algorithms, there is a need to mine additional functional plastid promoter motifs beyond those listed above. Experimental testing and identification of different PEP promoters will increase the availability of resources for developing new regulatory approaches in chloroplast engineering.

**Table 2:** Characterized plastid promoters and their strucutral components.

Type	Promoter*	Sequence^#^	Structural components	References
PEP				
	P*rbcL* (T)	-35 to +9	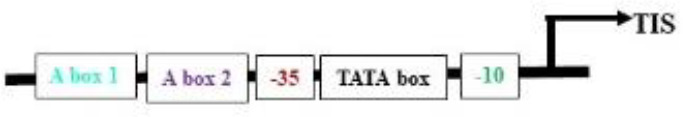	Shiina et al., 1998
	P*psbD* (T)	-78 to +28	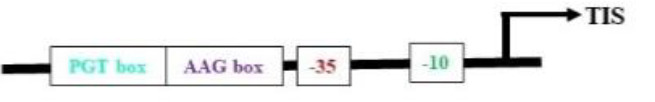	Allison and Maliga, 1995
	P*psbA* (T)	-42 to +9	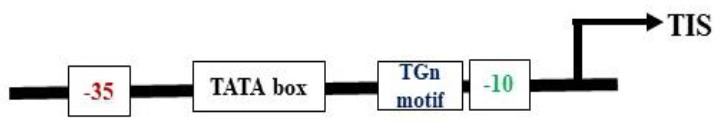	Hayashi et al., 2003
	P*rrn*P1 (T)	-64 to +17	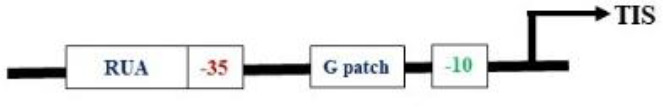	Suzuki et al., 2003
NEP				
Type-I- Ia	P*rpoB*-345 (T)	-15 to + 5	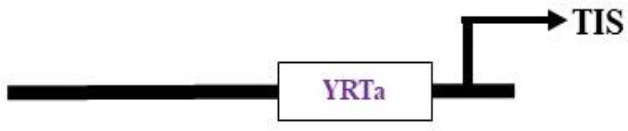	Liere and Maliga, 1999a
	P*rpoB*-300 (A)			
	P*rpoB*-147 (M & B)			
	P*aacD*-129 (T)			
	P*aacD*-251 & 172 (A)	-99 to +23	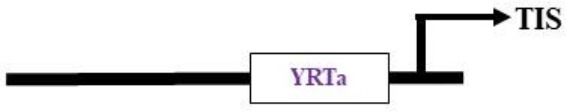	Hirata et al., 2004
Ib	P*atpB*-289 (T)	-43 to + 38	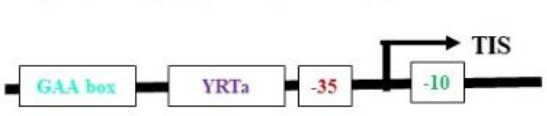	Kapoor and Sugiura, 1999
	P*atpB*-318 (A)			
	P*atpB*-601 (M)			
	P*atpB*-593 (B)			
Type- II	P*clpP*-53 (T)	-5 to +25	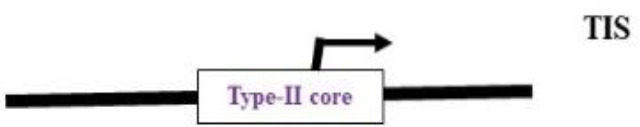	Sriraman et al., 1998

*rpoB, RNA polymerase b-subunit; *aacD*, Acetyl-CoA carboxylase encoding β-carboxyltransferase subunit; *atpB*, β-subunit of ATP synthase; *clpP*, Catalytic subunit of the protease Clp; *rbcL*, Rubisco large subunit; *psbD*, D2 protein of PSII; *psbA*, D1 protein of PSII; T, Tobacco; A, Arabidopsis; M, Maize; B, Barley; ^#^Sequence relative to transcription initiation site (TIS).

**(b) NEP promoters**

Nuclear-encoded polymerase (NEP) promoter activity has been identified at transcription initiation sites for several plastid genes in plants (**Table 2**). This activity is capable of driving transgene expression preferentially in non-green plastids, as found in fruits and tubers (Zhang et al., 2012; Valkov et al., 2011). The NEP promoters analyzed thus far are categorized into three types based on their sequence properties. Type-I promoters, characterized by a conserved YRTA-motif critical for promoter recognition, are located upstream of the transcription initiation site (+1) at -15 to +5 for P*rpoB*-345 and at -99 to +23 for P*accD*-129 in tobacco (Liere and Maliga, 1999a, b; Kahlau and Bock, 2008). These fall under the Ia-type category. Another subset, the Ib-type, carries a second conserved sequence motif (ATAGAAATAGAA) at ~18 to 20 bp upstream of the YRTA-motif (P*atpB*-289 at -43 to +38 in tobacco) (Kapoor and Sugiura, 1999). The type-II NEP promoter P*clpP*-53 from tobacco, which lacks the YRTA-motif, has been characterized and found to be primarily located downstream of the transcription initiation site at –5 to +25 (Sriraman et al., 1998a). The most notable feature of this promoter, studied in tobacco roots and amyloplasts of potato, is its capacity to lead expression with higher mRNA accumulation in non-green plastids (Zhang et al., 2012; Valkov et al., 2011). Lastly, there is a non-YRTa-type (Pc) promoter that shows NEP transcription activity at *rrn* operon sequences. However, the sites relevant for transcription initiation from Pc have yet to be identified (Baeza et al., 1991). These findings may contribute to the future development of chloroplast expression systems, which are anticipated to address the ineffective expression of transgenes in non-green chloroplasts.

**(c) 5’ Regulatory UTRs**

The 5′ UTRs of plastid genes contain a conserved motif, such as the Shine–Dalgarno sequence or ribosomal-binding site (RBS) (AGGAGG), which regulates mRNA stability (**Figure 2**). The best-studied 5’ UTR is that of *psbA* (encoding D1 protein of PSII) from tobacco, which includes three conserved RBS sites, an AU-box, and a 5’ stem-loop structure. Removal of the first 17 bp in the 5’ stem-loop reduces mRNA levels by 50% and translation efficiency by 75% (Staub and Maliga, 1994b). The structure of the 5’ UTR of *rbcL* (encoding Rubisco large subunit) contains a single RBS site and two 5’ stem-loops, which are essential for increased mRNA stability. Mutations in the first 30 bp downstream of the translation initiation site reduce translation efficiency by 35-fold (Kuroda and Maliga, 2001b). The 5’ UTR of *atpB* (encoding ATP synthase β-subunit) lacks a conserved RBS sequence, and a mutation in the -25 to +18 region was shown to reduce translation by more than 20% (Hirose and Sugiura, 2004). Similarly, the *psbN* (encoding N-subunit of PSII) 5’ UTR, which also lacks a conserved RBS site, requires an unknown RNA-binding protein to initiate translation (Kuroda and Sugiura, 2014). The 5’ UTR of *rps2* (encoding ribosomal protein S2) operates through an opposing mechanism, in which the binding of an RNA-binding protein limits translation efficiency by 13–20 fold (Plader and Sugiura, 2003). Finally, the *Escherichia coli* phage T7 gene (*T7g10*) and downstream box (DB) region are the most commonly used 5’ UTRs in plastid transgene expression. Protein expression levels have been shown to increase by up to 23% of the total soluble protein (TSP) when the expression is under the control of *T7g10* (Kuroda and Maliga, 2001a). Various studies have suggested that the combination of various ribosome-binding sites and their manipulations can be used to design and test new synthetic plastid 5’ UTRs that regulate gene expression (Drechsel and Bock, 2011).

**(d) 3’ Regulatory UTRs**

The most common features of 3’ UTRs include transcription termination, mRNA transcript stability, and cleavage of polycistronic mRNAs into monocistronic protein-coding sequences by stem-loops (Monde et al., 2000) (**Figure 2**). The 3’ UTRs of the *rbcL*, *rps16* (encoding ribosomal protein S16), and *psbA* genes are the most extensively used for plastid transgene expression. A study comparing the 3’ UTRs of *psbA* and *rpl32* (encoding ribosomal protein L32) in tobacco have shown a fivefold difference in mRNA abundance (*rpl32* > *psbA*) (Eibl et al., 1999). Similarly, a study comparing different 3’ UTRs, including those from plastid genes *rbcL*, *psbA*, *petD*, and *rpoA*, with the *E. coli rrnB* terminator, found that genes with the *psbA* and *petD* (encoding subunit IV of *cyt b6f)* 3’ UTRs exhibited two to three times higher transcript abundance compared to those with the *rbcL* and *rpoA* (encoding RNA polymerase α-subunit) 3’ UTRs (Tangphatsornruang et al., 2011). The examples above provide interesting insights, and future research may reveal more about the interplay between 3’ UTRs and transgenic plastid expression.

**(e) Plastid intercistronic expression elements**

**Figure 3:**
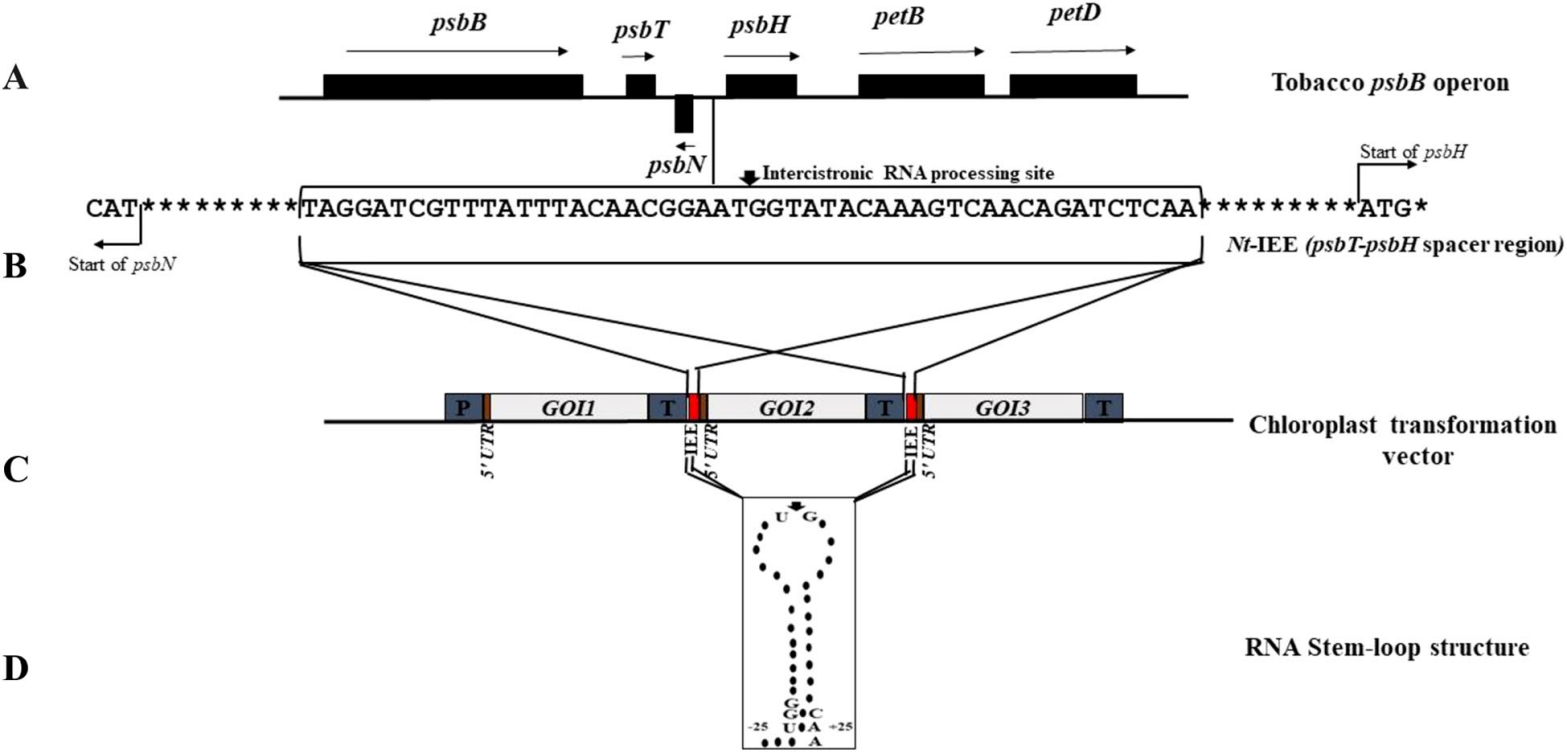
Intercistronic expression elements (IEEs) in the tobacco *psbB* operon. **(A)** Structure of the *psbB* operon with arrows above the genes indicating expression from left to right and arrow below the gene (*psbN*) indicating expression in the opposite direction. **(B)** Partial sequence of the *psbT–psbH* (*Nt*-IEE) (Zhou et al., 2007) spacer region from tobacco, used as a putative processing sequence in a plastid transformation vector. The position of the bold-headed arrow indicates the intercistronic RNA processing site (* indicates the next proceeding base). **(C)** A typical characteristic arrangement of IEEs inserted between the three cistrons (*GOI*: Gene of interest) to support plastid multicistron transgene expression. **(D)** Putative RNA stem–loop structure showing endonucleolytic cleavage site (at the bold headed arrow) for intercistronic processing of the operon.

The majority of chloroplast genes are expressed as polycistronic mRNAs. Primary transcripts in plastids are post-transcriptionally processed in the 5’-3’ direction into monocistronic or oligocistronic mRNAs through a complex series of maturation steps mediated by nuclear-encoded prokaryotic-type ribonucleases (Sakamoto et al., 1994). Stem-loop-like secondary structures within the 5’ and 3’ UTRs of plastid mRNA serve as recognition elements for RNA processing enzymes and act as protective elements against RNA degradation (Hayes et al., 1996; Stern and Gruissem, 1987). The transcripts from most plastid operons undergo essential intercistronic processing (RNA cleavage) to form monocistronic units, which in turn makes translation more efficient. One example is the *psbE* operon, which is transcribed as a single tetracistronic mRNA containing the *psbE, psbF, psbL* and *psbJ* genes for photosystem II (Carrillo et al., 1986; Willey and Gray, 1990). Stacking of multiple transgenes is often employed in chloroplast biotechnology and remains one of the unique attractions of chloroplast transformation technology, allowing the expression of multiple genes under a single promoter. IEEs, which are small sequence elements that mediate intercistronic cleavage (Zhou et al., 2007), are effective for stacking multiple foreign genes in operons and help to expand the range of applications for transplastomic technology. Two intercistronic cleavage sites were identified in the tobacco *psbB* operon of photosystem II, located at the central position of a putative RNA stem-loop secondary structure: *psbT-psbH* and *psbH-petB* (**Figure 3**). *In vivo* testing of these two elements, placed between two transgenes, revealed that only the complete stem-loop structure surrounding the *psbT–psbH* processing site conferred expression of the downstream cistron and thus acted as a functional IEE (Zhou et al., 2007). The IEE (50 bp) greatly simplifies the vector construction for co-expression of multiple genes and eliminates the need for individual gene promoters, reducing the risk of unusual recombination events and partial genomic deletions. The use of IEEs in tobacco and tomato chloroplasts has been evaluated in the construction of a synthetic operon for the expression of homogentisate phytyltransferase (*SyHPT*), tocopherol cyclase (*SyTCY*) and c-tocopherol methyltransferase (*AtTMT*), which are genes involved in the vitamin E (tocochromanol) biosynthesis pathway. This experiment resulted in a tenfold increase in the accumulation of transgene-derived foreign proteins compared to levels without IEEs (Lu et al., 2013). Another significant study in *C. reinhardtii* identified *psbN*-*psbH* and *tscA*-*chlN* as functional intercistronic regions and their role in the expression of foreign genes was identified (Macedo-Osorio et al., 2018).

**(f) Pentatricopeptide repeat binding sites**

**Figure 4:**
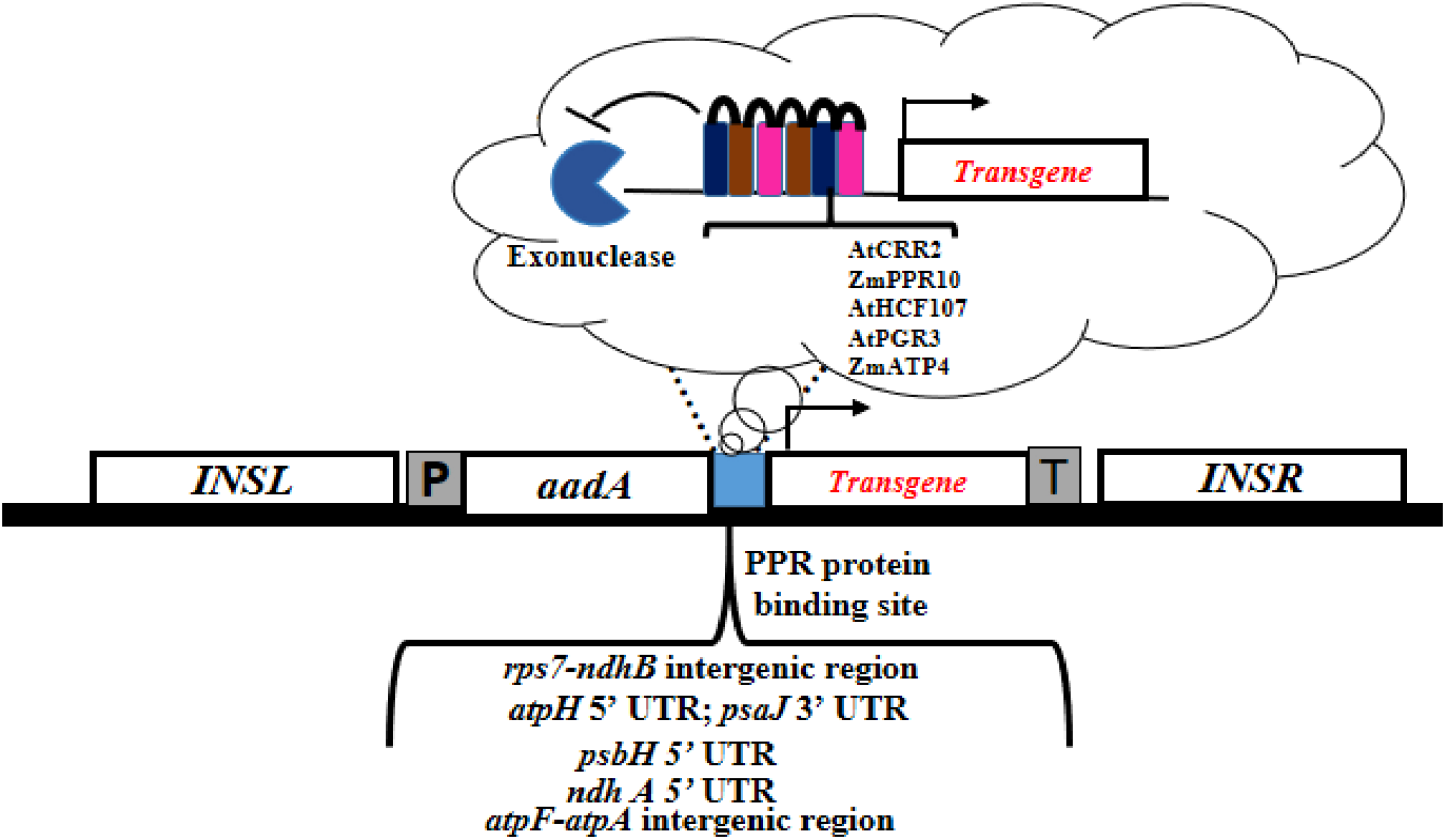
Experimental strategy for pentatricopeptide repeat (PPR) protein-mediated transgene expression in chloroplasts. Schematic overview of different PPR proteins and their binding sites. The incorporation of PPR protein binding sequences upstream of the transgene will enhance its expression by blocking exonuclease-mediated RNA degradation and increasing translational efficiency (in enlarged view).

PPR proteins are sequence-specific RNA-binding proteins involved in RNA maturation in plastids (Barkan et al., 2012). PPR proteins are of particular interest to chloroplast biotechnologists due to their potential for engineering the regulated expression of transgenes in chloroplasts. This regulatory mechanism involves the interaction between a nuclear-controlled variant of the PPR protein and a cognate binding site upstream of a chloroplast transgene, enabling its regulated expression. PPRs interact with RNA in a repeat-to-nucleotide binding mode via a superhelical RNA-binding surface that contains a lengthy stack of repeating motifs (Zhou et al., 2017) (**Figure 4**). Engineered PPR proteins can be generated by altering the PPR sequence or redesigning the protein based on the consensus PPR motif for custom RNA binding (Yan et al., 2019). An ethanol-inducible on/off switch for regulating chloroplast transgenes has been developed based on the characterized maize PPR10, which stimulates *atpH* expression by blocking 5′→3′ exoribonucleolytic degradation of mRNA (Rojas et al., 2019). This system has enhanced the translational efficiency of GFP and increased transgene expression in leaf tissues (Yu et al., 2019). Another PPR protein, chlororespiratory reduction 2 (CRR2), was employed to express vaccine-candidate genes against the human papillomavirus (HPV). Immunological studies showed that a post-transcriptional expression element (*ndhB* sequence) was helpful in expressing L1 proteins, though the yield was low (Legen et al., 2023). Overall, PPR protein-mediated strategies provide one of the few options available to chloroplast biotechnologists to regulate and optimize the expression of foreign genes (genes of interest) in chloroplasts.

**(g) Elements for inducible expression**

**Figure 5:**
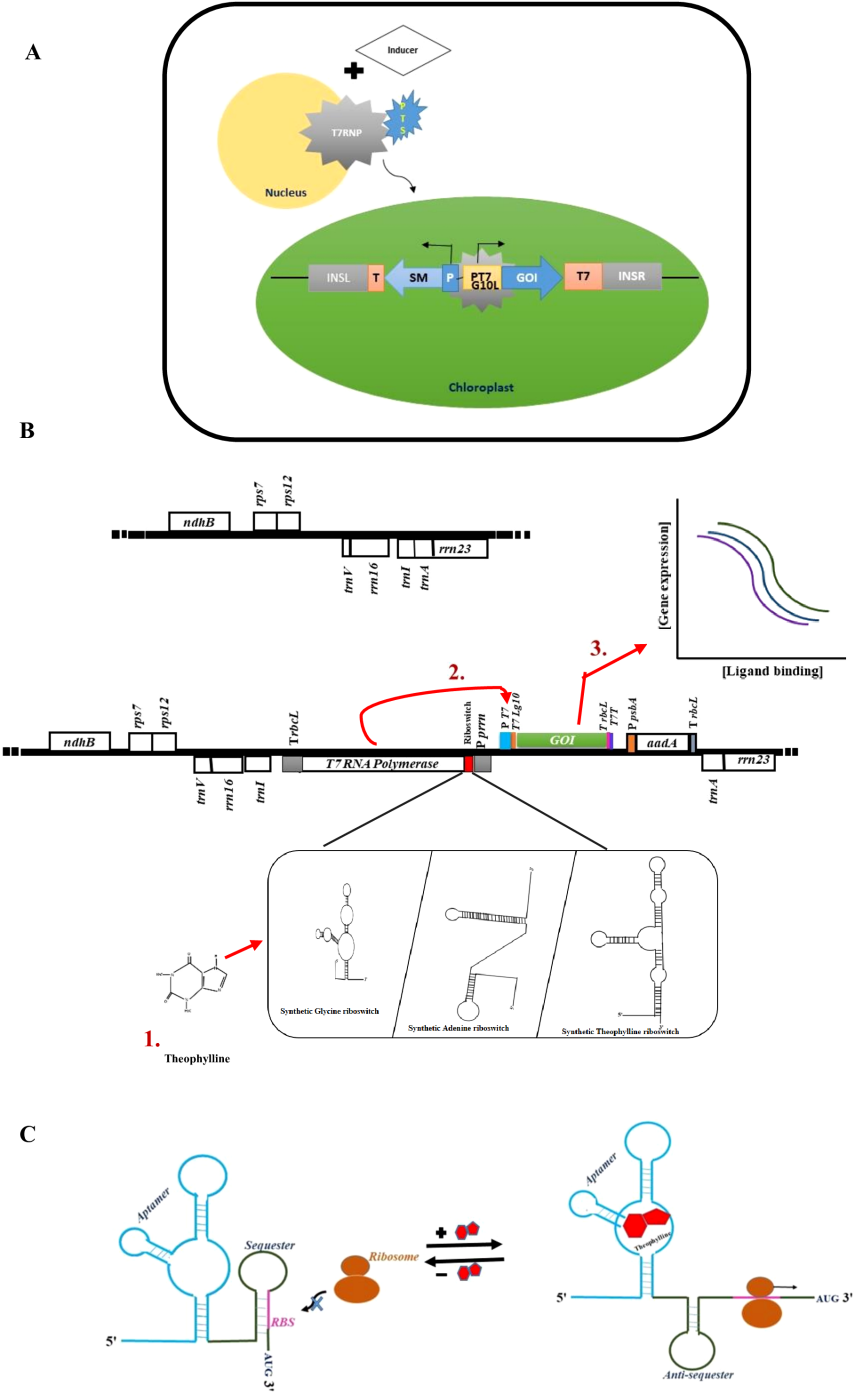
Different approaches for inducible transgene expression in plastids. **(A)** A nuclear gene- encoded T7 RNAP (RNA polymerase) fused to a plastid targeting sequence (PTS) for trans-activation in plastids expressed under control of an inducible promoter. The application of the inducer leads to the import of T7 RNAP into the chloroplasts, activating the transcription of plastid transgenes. **(B)** RNA amplification-enhanced riboswitch (RAmpER) mediates inducible transgene expression in the genome. A representative physical map of the plastid transformation vector showing the induction by theophylline (1,3-dimethylxanthine) (step 1), which activates the T7 RNA polymerase gene in the presence of a translational riboswitch (step 2). This is sufficient to trigger immense transcription of the gene of interest (*GOI*), driven by the T7 promoter (step 3). **(C)** In the absence of theophylline, translational control of gene expression by the synthetic theophylline-responsive riboswitch is facilitated by a folded structure that sequesters the ribosome binding site (RBS) in the mRNA transcript. In the presence of theophylline, the riboswitch adopts a conformational change at the aptamer, promoting the translation mechanism by releasing the RBS.

Overexpression of foreign proteins may result in severe mutant phenotypes. This can occur due to the complete depletion of gene expression capacity or interference of the recombinant protein(s) with chloroplast biogenesis. Pigment deficiencies and impaired photosynthetic activities typically result in negative effects during chloroplast transgene expression (Lössl et al., 2003; Hennig et al., 2007). Unfortunately, studies on endogenous mechanisms that control gene expression in chloroplasts have been limited. It is highly anticipated that an inducible transgene expression system will be developed to facilitate the accumulation of foreign proteins in response to an external stimulus. Nuclear control for regulating plastid transgene expression was initially investigated in tobacco. This approach uses a plastid-targeted T7 RNA polymerase, encoded in the nucleus, with its transcription regulated by ethanol-inducible nuclear promoters (Lössl et al., 2005) (**Figure 5A**). Two other inducible techniques that rely on nuclear control have been created. A hybrid transcription system was developed to utilize a eubacterial promoter, under which the transgene is positioned for expression. A chimeric transcription sigma factor that interacts with the PEP is required for this action (Buhot et al., 2006). The use of nuclear-encoded and plastid-targeted site-specific recombinase (Cre) for excision of an expression-blocking sequence that drives transgene expression has also been developed (Tungsuchat et al., 2006). These two methods involve the nuclear control for plastid transgene expression, which nullifies the advantage of abolishing transgene containment conferred by maternal inheritance—one of the most striking features of plastid engineering technology (Barkan and Goldschmidt-Clermont, 2000).

The development of plastid-only inducible systems is highly desirable for the direct regulation of all components from the chloroplast genome. Two such systems have been reported: an external control system for plastid gene expression that responds to chemical induction by isopropyl-β-D-thiogalactopyranoside (IPTG), which regulates transcription via a modified plastid promoter containing a LacI-binding site from *E.coli* (Mühlbauer and Koop, 2005), as well as the development of riboswitches, which are regulatory ribonucleic acid sequences (cis-acting mRNA elements) that bind to ligands and directly affect gene expression by forming alternative structures. Characterization of synthetic riboswitches for inducible gene expression has shown that their function can be altered by sequence modification for translational regulation, as plastids do not have effective transcription termination mechanisms. The successful design and evaluation of different synthetic chloroplast riboswitches from *E.coli* [e.g., thiamine pyrophosphate (TPP) and synthetic theophylline (theo) riboswitches] were reported (Verhounig et al., 2010). In addition, two other transcriptional “on” switches [the glycine (gly) and the adenine (ade) switches] from *B. subtilis* with modified Shine-Dalgarno (SD) or anti-Shine-Dalgarno (anti-SD) structures were developed to facilitate translational regulation (Verhounig et al., 2010). A synthetic theophylline switch (s.theo-RS) was also functionally verified for its exceptional gene regulation ability in tobacco chloroplasts (Verhounig et al., 2010). The challenge of relatively low transcription levels with riboswitches was addressed by adding a bacteriophage T7 RNA polymerase-mediated RNA amplification step, resulting in a new RNA amplification-enhanced riboswitch (RAmpER) (**Figure 5B**) (Emadpour et al., 2015). Riboswitches, mostly located in the 5’ UTR, regulate gene expression by either directing the switching sequence to form a short stem-loop structure that aborts transcription, or by exposing/concealing the Shine-Dalgarno sequence to control translation. In some eukaryotic mRNAs, the TPP riboswitch also regulates 3’ end splicing (Barrick et al., 2004) (**Figure 5C**). Improved inducible expression systems offer expanded possibilities regarding the application of transplastomic technology. Recently, RAmpER was successfully employed in the regulation of astaxanthin synthesis (Agrawal et al., 2022) and antimicrobial peptides (AMPs) (Hoelscher et al., 2022) in tobacco.

#### 2.1.5 *In vivo* vector assembly

The development of a transformation vector that targets the *trnf*M and *trn*G genes of the tobacco chloroplast genome using *in vivo E. coli* cloning (iVEC) technology is another significant advance in chloroplast engineering. Wu et al. (2017) developed a simple and seamless method to construct a complete plastid transformation vector in a single step with five DNA fragments. PCR products comprising four insert DNA fragments and a linearized vector, all with homologous short sequences at both ends, are co-transformed into competent *E. coli* cells. The homologous recombination events in *E. coli* allow the inserts and vector backbone to recombine *in vivo*, generating a functional plastid transformation vector. The iVEC-assembled transformation vector has been used to generate transplastomic plants, which exhibit foreign protein accumulation levels of 9% of total soluble protein. Recently, modular cloning has taken advantage of type-IIS restriction endonucleases that cut dsDNA at a specified distance away from the recognition sequence, creating custom overhangs that are efficiently involved in the assembly of genetic elements in a one-step reaction. The Golden Braid (GB) modular cloning procedure has been described to design and assemble DNA fragments (promoters, UTRs, and multigene operons) in plastid transformation vectors. This led to the establishment of a plastid expression vector for the formation of griffithsin using a set of modules that facilitate transformation in tobacco (Vafaee et al., 2014). Occhialini et al. (2019) developed the fast and flexible chloroplast engineering toolkit “MoChlo” by assembling synthetic operons of various sizes and determined the efficiency of assembly. This library consists of 128 standardized chloroplast-specific parts, which have been used successfully to generate chloroplast transformation vectors containing up to seven transcriptional synthetic operons in a single vector (10.6-kb). The main objective of establishing the modular cloning kits is to create a repository of reusable genetic components necessary for engaging chloroplast engineering.

### 2.2 Chloroplast transformation methods

#### 2.2.1 Classical gene delivery methods into plastids

The two most common methods for delivering genes into plastids are the polyethylene glycol (PEG)-mediated method and particle bombardment. The less commonly used approach for gene delivery into the plastome involves PEG-mediated transformation, which requires enzymatic removal of the cell wall to obtain protoplasts, followed by exposing the protoplasts to purified DNA in the presence of PEG (Koop et al., 1996; O'Neill et al., 1993). However, the lack of effective protoplast regeneration protocols for producing plastid-transformed commercial crops presents a challenge for this methodology, resulting in a reduced success rate. Biolistic technology has facilitated the direct delivery of foreign DNA directly into the plastome by crossing the physical barrier imposed by the double membrane of the chloroplast envelope (Sanford, 1990). Particle bombardment, the method of choice for engineering various crops, remains extremely versatile in facilitating the generation of transplastomic lines. This is accomplished by delivering plastid transformation DNA constructs into plant cells by linking the genetic material to microsized metal particles (usually gold or tungsten), followed by high-velocity deployment to explants (Klein et al., 1987; Kohli et al., 2001) (**Figure 1B**). A wide variety of cell types can be targeted efficiently for foreign DNA delivery, including leaf cuttings (Svab et al., 1990), calli (Wang et al., 2018), hypocotyls (Narra et al., 2018), and root explants (Ruf et al., 2019) (**Table 1**). The frequency with which transformed plants can be recovered after bombardment depends on the efficiency of regeneration of explants under selective antibiotic culture conditions (Altpeter et al., 2005). One of the major advantages of this method is that it does not rely on biological constraints (cellular and physiological factors that limit DNA uptake and stable integration) or host limitations (regeneration and species-specific transformation barriers) (Christou et al., 1991). Particle bombardment uses a minimal DNA expression cassette flanked by homologous chloroplast sequences to coat the metal particles and carry out the transformation (Fu et al., 2000). The major obstacle in engineering the plastid genome is the long process of selection for generating homoplasmic transplastomic plants. This is due to the presence of 10–100 plastids, each with about 100 copies of the plastid genome, in one cell, which makes it difficult to achieve a homoplastomic state in a single attempt (Ho and Cummins, 2005) (**Figure 1C**).

#### 2.2.2 Chloroplast transformation by *Agrobacterium tumefaciens*

A few attempts have been made to optimize *Agrobacterium*-mediated transformation to efficiently introduce the Ti-plasmid carrying the genes into chloroplasts (**Table 3**). Initial attempts at *Agrobacterium* mediated transformation in chloroplasts resulted in chloramphenicol-resistant tobacco plants after cocultivation. Although these results were consistent with the delivery of the *cat* gene encoding chloramphenicol acetyltransferase contained in the plastid transformation vector, the process was hindered by difficulties in obtaining stable transgenic lines (De Block et al., 1985). This may be due to a lack of flanking chloroplast targeting sequences in the vector, which are necessary for the integration and maintenance of foreign DNA sequences. In a different study, the T-DNA segment of a binary vector comprised of 5S rRNA and a tRNA was used to generate kanamycin-resistant clones. Chloroplast transformation events were characterized by single crossover events, which resulted in unstable cointegrate structures (Venkateswarlu and Nazar, 1991). Neither of these two experiments provided a reproducible methodology for chloroplast engineering, despite the unexpected expression of marker genes. Matsuoka and Maliga (2021) made a successful attempt to re-engineer the virulence proteins VirD2 and VirE2 for targeted T-DNA delivery into tobacco chloroplasts. The authors provided the evidence for the direct export of the phiC31 phage site-specific integrase (*Int*) protein fused with the Rubisco small subunit transit peptide (TP) at the N-terminus and the VirF (F) T4SS signal at the C-terminus.

**Table 3:** Summary of different delivery methods adopted for plastid transformation.

Transformation method	Targeted species	▴ Advantages & • limitations	References
Biolistics	*Nicotiana tabacum & many other species*	▴High transformation efficiency •Tissue damage •Costly for gold particles	Maliga, 2004; Bock, 2015
PEG	*Nicotiana tabacum*	▴Low cost •Low transformation efficiency	Golds et al., 1993
Agro-infiltration	*Nicotiana tabacum*	▴Easy to handle & low-cost •Host limitation	Matsuoka and Maliga, 2021
Nanoparticle-mediated gene transfer	*Eruca* *sativa* *Nasturtium* *officinale* *Nicotiana* *tabacum* *Spinacia* *oleracea* *Arabidopsis thaliana*	▴Low cost • Difficulty in preparation •Possibility of toxicity	Kwak et al., 2019
CPP/CTP	*Nicotiana tabacum* *Oryza sativa* *Hibiscus cannabinus*	▴Time-saving & useful for recalcitrant crops •Low efficiency	Odahara et al., 2022
	*Arabidopsis thaliana*		Oikawa et al., 2021
Episomal engineering	*Solanum tuberosum*	▴Gene amplification without integration into native plastid genome •Hard to maintain for next generations	Occhialini et al., 2021
Horizontal genome transfer (HGT)	*Nicotiana tabacum* & *Nicotiana glauca*	▴Suitable for recalcitrant species • Grafting site should be recultured	Lu et al., 2017
Linear DNA fragments	*Nicotiana tabacum*	▴Low cost without need of compact vector • Low efficiency in multiple gene expression •Accurate amplification & sequencing of fragments is necessary	Ren et al., 2022

#### 2.2.3 Nanoparticle-based plastid engineering

Novel options for improving chloroplast biotechnology are being opened up by developments in nanotechnology. Through the penetration of plant cell walls, cell membranes, and organellar double envelopes, nanomaterials provide a viable means of delivering transgenes (Ganesan et al., 2018; Newkirk et al., 2021) (**Table 3**). The general influence of nanoparticle properties such as size and coating on their uptake and transport processes has been investigated successfully (Avellan et al., 2019). The hydrophilic quantum dot (QD) with β-cyclodextrin molecular baskets conjugated with targeting peptides (RbcS: Rubisco small subunit 1A) allowed the targeted delivery of biochemical cargoes into *Arabidopsis* chloroplasts (Santana et al., 2020). Nanoparticles can be functionalized with biodegradable polysaccharides such as chitosan to form DNA-nanoparticle complexes that are capable of electrostatic cellular import and chloroplast delivery. A notable example of DNA-chitosan complex for chloroplast transformation was studied in *Eruca sativa, Nasturtium officinale, Nicotiana tabacum*, and *Spinacia oleracea* chloroplasts for transient expression of yellow fluorescent protein (YFP) through chitosan-wrapped single-walled carbon nanotubes (CS-SWNTs) (Kwak et al., 2019). In this study, positively charged CS-SWNTs were complexed with negatively charged plasmid DNA (pDNA), forming a net positive nanocomplex that migrates into chloroplasts. Once in the chloroplast, the pDNA is thought to disassemble from the CS-SWNTs in the weakly alkaline environment (pH ~8), resulting in a transformation frequency of approximately 88%. This success demonstrates that nanotechnology holds great promise as a platform for chloroplast transformation and should be further optimized for applying this technology to other economically important crop species.

#### 2.2.4 Clustered cell-penetrating and chloroplast-targeting peptides mediated plastid transformation

Efficient peptide-mediated pDNA delivery to plastids can be achieved through proper linkage to cell-penetrating peptides (CPP) and chloroplast-targeting peptides (CTP) (**Table 3**). A transient plastid transformation technique has been established in leaves of *A. thaliana* and *N. benthamiana* and in mature green tomato fruits, using formulated pDNA/CTP/CPP complexes with similar efficiencies (Thagun et al., 2019). Electrostatic interaction at optimal amine/phosphate (N/P) ratios between pDNA and cationic CTP (KH9-OEP34) enables the condensation of pDNA and its effective delivery to plastids. Decreased transformation efficiency has been noted at higher N/P ratios, which may be due to the slow dissociation of pDNA/CTP complexes once they are delivered into plastids. In contrast, lower N/P ratios may cause destabilization and premature release of bound pDNA before reaching the target (Thagun et al., 2019). The addition of BP100 (CPP), a positively charged, membrane-binding agent, to pDNA/CTP complexes resulted in enhanced plastid transfection efficiency. Oikawa et al. (2021) developed a simple infiltration method to introduce a complex solution into intact plant cells for studying plastid-localized transgene expression. Thagun et al. (2022) introduced a high-throughput peptide-mediated foreign DNA delivery platform by spraying the leaf surface. The translocation of the CPP/CTP/pDNA complex is thought to be controlled by a stomata-dependent uptake mechanism. Odahara et al. (2022) recently studied peptide-mediated plastid transformation in tobacco, rice, and kenaf to transport foreign DNA into plastids. A fusion peptide (KH-AtOEP34) was generated by combining a polycationic DNA-binding peptide (KH) and a plastid-localizing signal of outer envelope membrane protein-OEP34/TOC34 (AtOEP34) to integrate a foreign DNA construct into the chloroplast through homologous recombination. The resultant transformants were selected for resistance to spectinomycin/streptomycin and were found to be fertile. The integrated foreign genes were successfully transmitted to the T1 generation. Another study characterized the route and tracked the movement of DNA/CTP/CPP complexes into chloroplasts of *Arabidopsis thaliana* (Oikawa et al., 2021). Gene delivery from the extracellular space to the chloroplast stroma was detected through fluorescent labeling and confocal laser scanning microscopy (CLSM), which occurred 6hrs after infiltration. Based on these successful attempts, peptide-mediated plastid transformation could serve as a robust transformation method in economically important agricultural plant systems.

#### 2.2.5 Episomal plastid engineering

**Figure 6:**
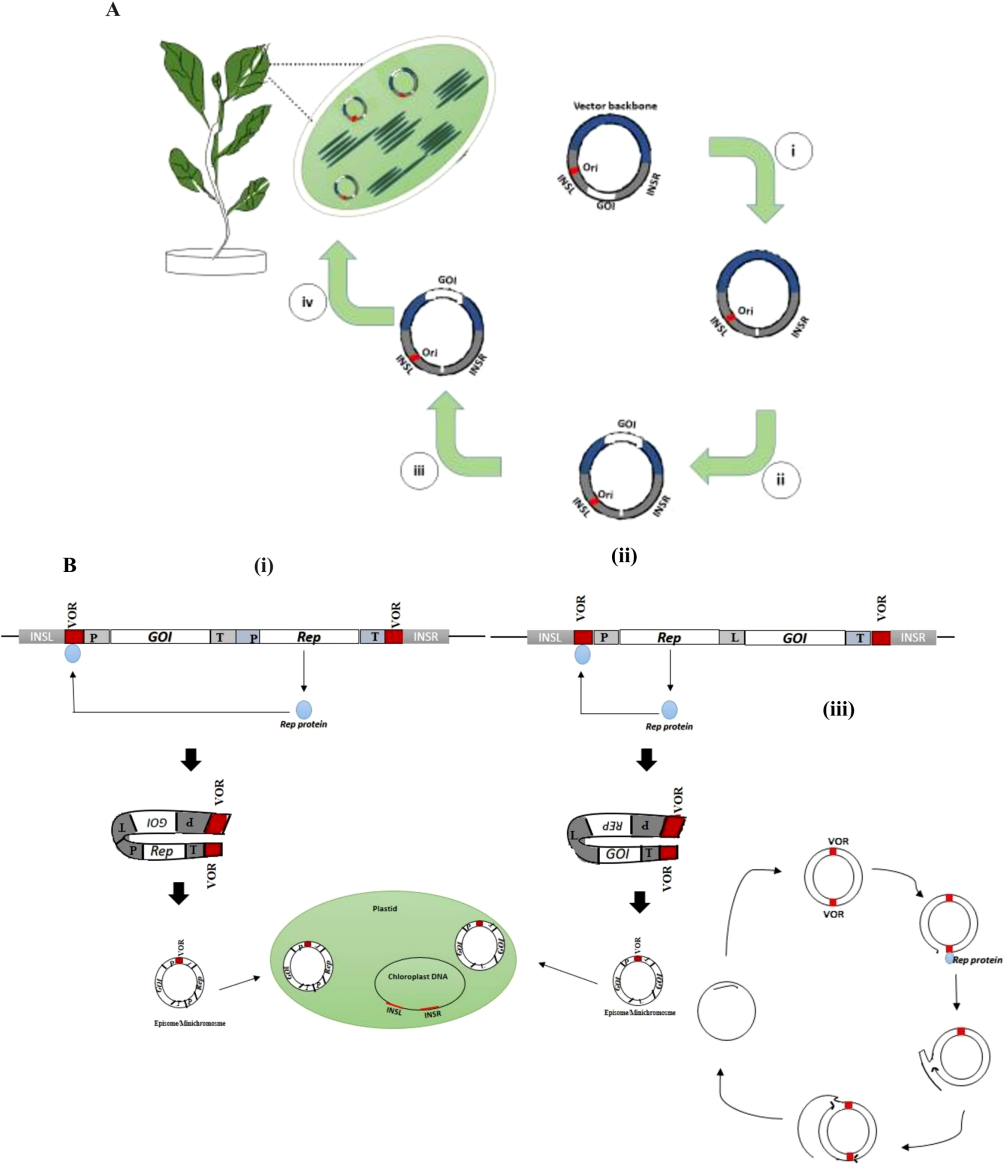
Episomal vector system for plastid transformation. **(A)** Plastid genetic engineering using a non-integrating episomal synthetic plastid vector, i.e., mini-synplastome (Occhialini et al., 2021). (i) A chloroplast-specific integration vector is used for transformation and selection of transplastomic lines without vector integration; (ii) Screening and isolation of plasmids from *E. coli* by back-transformation with total genomic DNA from transformed lines containing episomes; (iii) Construction of synthetic plastome with the gene of interest (GOI) for transforming chloroplasts; (iv) Mini-synplastomes as independent replicating molecule carrying the GOI. Insertional sequence left (INSL), insertional sequence right (INSR), homologous arms (Ash grey), the viral origin of replication (VOR), Ori (red), vector backbone (Blue), and the GOI (white) are indicated in the plasmids. **(B)** A design illustrating the episome replicating in the transplastomic plant generated using the episomal vector. (i, ii) Plastid-targeting vectors with the VOR, replication initiator protein (Rep), and GOI with flanking sequences, allows the target transgene insertion into chloroplast without integrating into native plastid genome. (iii) Mechanism of Rep-mediated recognition of a specific VOR sequence which initiates a specific nick to generate a ssDNA that can be converted to circular dsDNA.

Over three decades of progress in plastid engineering, the effective integration of transgene constructs into the plastome has relied on the mechanism of homologous recombination (HR). Now, in the age of synthetic biology, chloroplast biotechnology provides a dynamic platform for the one-step introduction of metabolic pathways in plants. However, the ultimate goal is to create marker-free transplastomic lines by choosing an alternative to the HR mode of chloroplast engineering. Episomally replicating synthetic plasmids may provide an interesting platform, with the ability to express transgenes in plastids without integrating vector sequences into the native plastid genome (Staub and Maliga, 1994) (**Table 3**). Persistence across multiple generations even when the selection pressure is removed is a key determinant for the commercial application of episomal plastid engineering. In this context, the design of the mini-synplastome, a novel synthetic episomal vector engineered using NICE1 ( *Nicotiana* plastid extrachromosomal element) (Staub and Maliga, 1994a) and dinoflagellate *ori* (origin of replication) (Min et al., 2015), has been pursued to explore possible methods for plastid transformation in potato plants without disturbing the native plastid genome (Occhialini et al., 2021). Shuttle vectors, developed in this study by inserting a selection and visible reporter gene cassette (*aadA* and *smGFP*) that was flanked by the vector backbone instead of homology arms, were shown to be capable of replication in *E. coli* and able to generate transplastomic plants. This work also demonstrated the possibility of designing an episomal construct in which the desired transgenes are incorporated through HR, while the selection process is carried out from a non-integrating region of the episome, leading to homoplasmy (**Figure 6A**). The selective pressure could be removed, enabling the gradual elimination of the episomal vector, resulting in marker-free transplastomic lines. A new method for transgene expression in chloroplasts has been developed using a ‘minichromosome’, a physically independent and amplifying entity. This method offers an alternate strategy for effective foreign gene expression in plastids by utilizing the replication mechanism of gemini viruses. The whole process relies on the helper protein ‘Rep’ (replication initiator protein), which mediates the amplification of transgene DNA by initiating replication via recognition of specific ‘VOR’ (viral origin of replication) sequences flanking it (**Figure 6B**). Further studies are needed to analyze the impact of minichromosome-induced phenotypes on progeny (Jakubiec et al., 2021).

#### 2.2.6 Horizontal gene transfer

Horizontal gene transfer (HGT), the process of exchanging genetic material from donor to recipient by asexual means, is a plausible mechanism between sexually incompatible species. Genetic experiments have revealed that the simple method of grafting allows the introduction of entire organelles (mitochondria and chloroplasts) and/or their genomes to cross non-transformable species barriers (Stegemann et al., 2012) (**Table 3**). This is achieved by cell wall thinning and *de novo* formation of plasmodesmatal connections at the graft union of stock and scion cells. The resulting outcome of horizontal transfer is different in chloroplasts, which do not normally undergo recombination, compared to plant mitochondria, which frequently recombine to form mosaic genomes (Thyssen et al., 2012; Stegemann et al., 2012). The grafting procedure, which facilitates the horizontal transfer of the plastid genome across the graft junction between *Nicotiana tabacum* and nicotine-free tree species *Nicotiana glauca*, led to the recovery of homoplasmic transplastomic *N. glauca* at a higher frequency (Lu et al., 2017). Several events have been reported in which HGT occurs through the cell-to-cell transfer of entire organelles with encapsulated genomes (Hertle et al., 2021). Overall, the HGT method is effective for transforming plastids from neighboring cells, enabling chloroplast transformation in species with low efficiencies, and thereby facilitating biotechnological applications.

#### 2.2.7 Plastid transformation with fragmented DNA

Construction of plastid transformation vectors has become cumbersome for the expression of multiple genes, despite the advances of new application tools. A low-cost, cloning-free method will be one of the best choices for chloroplast genome transformation with a single or few fragments (**Table 3**). One such system has been validated using multiple linear DNA fragments (Ren et al., 2022). This strategy relies on homologous recombination between DNA fragments that share homologous sequences (HS) at their ends, mediated by the endogenous plastid recombination machinery. The fragments that are delivered through particle bombardment are integrated into the plastid genome. The results have shown homologous sequences as short as 200 bp are sufficient for proper integration, and this method has been successfully employed to introduce a phage lysin-encoding gene (*plyGBS)* and a long operon with *AtVDE* (encoding violaxanthin de-epoxidase in *Arabidopsis thaliana*) and *AtZEP* (encoding zeaxanthin epoxidase in *Arabidopsis thaliana*) into the tobacco plastid genome. However, for the construction of synthetic pathways involving transformation and expression of multiple genes, it is important to consider that longer stretches of homologous sequences at fragment ends can have a positive effect on the efficiency of fragment insertions (Ren et al., 2022).

## 3 Advanced techniques in plastid genome manipulation and engineering

### 3.1 Plastid genome-targeted base editing

**Figure 7:**
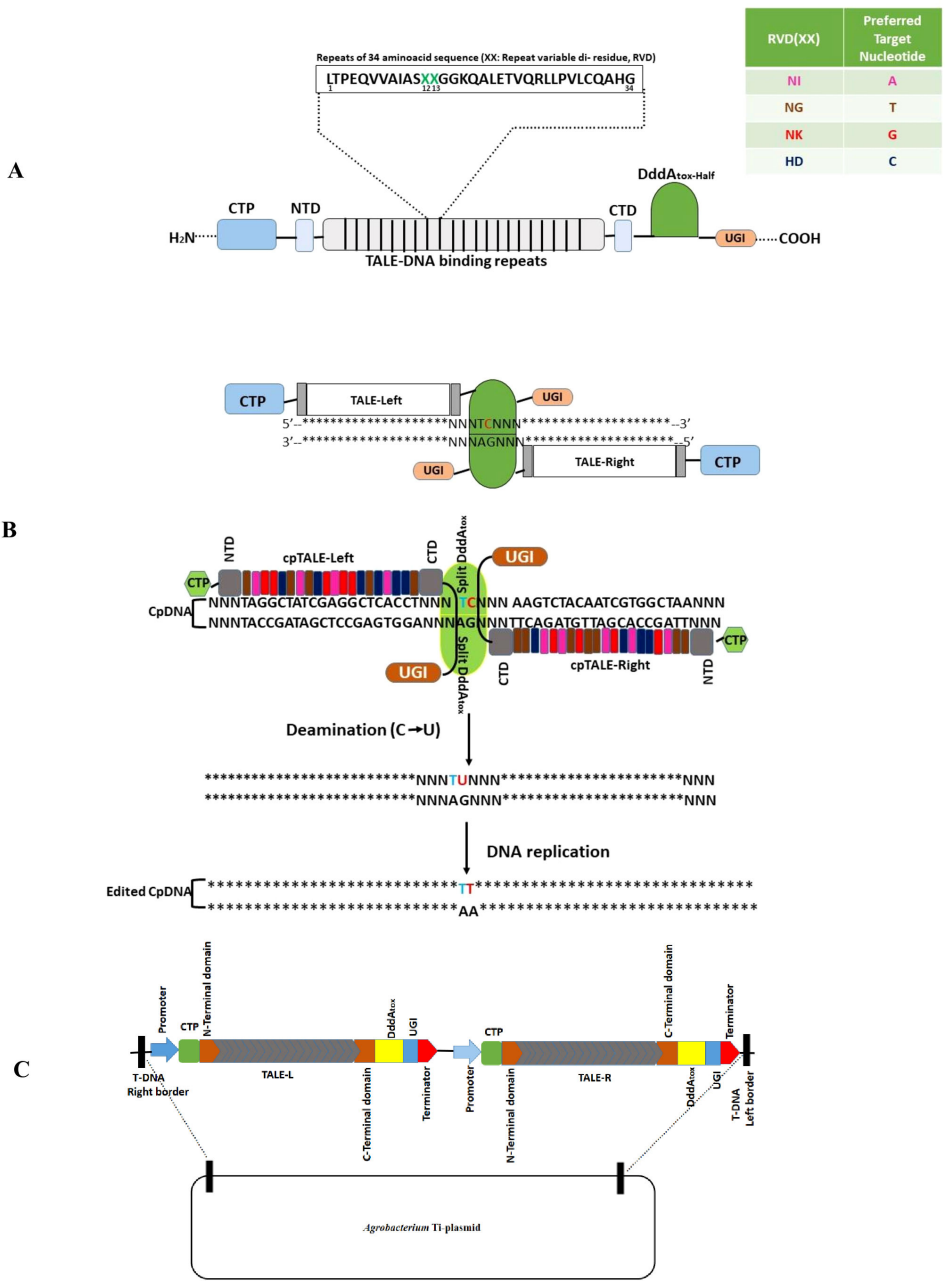
Mechanism of double-stranded DNA deaminase toxin A (DddA)-derived cytosine base editor (DdCBE) mediated chloroplast genome editing. **(A)** Structure of DNA binding repeats (TALE-array), with repeating units of 34 amino acids that each bind to one nucleotide. These units differ from one another at the 12^th^ and 13^th^ positions, the so-called repeat variable di-residues (RVD) which target a specific nucleotide (listed at the right) in the target DNA. The DdCBE repeating units normally vary in number up to 20. The N-terminal repeat unit of the TALE array protein binds the 5′ nucleotide of the target site (usually T will always be the first nucleotide). NTD and CTD are N-and C-terminal domains, respectively, flanking the DNA-binding TALE-array. A presequence (Chloroplast transit peptide, CTP) is added to the N-terminus for transporting the target proteins to chloroplast. The N- or the C-terminal part of DddAtox is fused to the C-terminus of the protein array. The complete complex binds to target DNA in a tail-to-tail manner, leaving a spacer region of approximately 14 nucleotides length where deamination reaction occurs, inducing a C-to-T conversion. **(B)** Mechanism of chloroplast DNA (CpDNA) editing involves the reconstitution of two halves of inactive DddAtox proteins in the chloroplast matrix to form active DddA cytidine deaminase base editor (DdCBE), connected with an uracil-DNA glycosylase inhibitory protein (UGI) (Asterisks* correspond to nucleotides). **(C)** Schematic representation of the tandem expression vector for *Agrobacterium-*mediated transformation.

Plant nuclear genome editing has revolutionized crop improvement by enabling genetic changes without the introduction of foreign genes. However, despite notable advancements in nuclear genome editing, only a few recent studies have led to significant breakthroughs in the genome editing of plant organelles (Kang et al., 2021). Targeted base substitutions are thought to be a superior way to make desired single nucleotide polymorphisms (SNPs) without disturbing other genes in the plastid genomes of land plants. To date, methods for C-to-T and A-to-G base editing have been reported. Double-stranded DNA deaminase toxin A (DddA)-derived cytosine base editors (DdCBEs) have been developed for organellar DNA C-to-T base editing in *A. thaliana* (Nakazato et al., 2021, 2022), lettuce, and rapeseed (Kang et al., 2021). These CRISPR-free, protein-only base editors are fused to two halves of the DddA active domain at the C-terminus, allowing them to recognize and come together on adjacent binding sites of the target DNA. The spacer sequence separating the two TALE (Transcription activator-like effector domains)-binding sequences are edited at respective positions (**Figure 7A**). To target the chloroplast genome, suitable chloroplast transit peptide (CTP) sequences were added to the N-terminus of the TALE monomers. To enhance mutation frequency, uracil-DNA glycosylase is inhibited by attaching a copy of uracil DNA glycosidase inhibitor (UGI) to the C-terminus of the DdCBEs, preventing the excision of uracil from the target DNA (Tan et al., 2022) (**Figure 7B**). The Golden Gate assembly system was used to construct custom-designed DdCBEs encoding plasmids to target *16S rRNA*, *psbA*, *psbB*, *atp6*, and *rps14* genes in chloroplast DNA and *atp6* and *rps14* genes in mitochondrial DNA. This led to high frequencies of C-to-T conversions in lettuce and rapeseed protoplasts by PEG-mediated transfection. Editing events were observed in calli derived from transfected protoplasts with frequencies of up to 38% (Kang et al., 2021). Recent studies have reported the establishment of homoplasmic (100% edited) lines in the T2 generation of *A. thaliana*, altered with C-to-T transition after the elimination of the introduced DdCBE transgene from the nucleus by Mendelian segregation. Some off-target editing events were observed near the targeted sites or far away in the plastid genome (Nakazato et al., 2021). Nakazato et al. (2021) referred to their use of plastid-targeted DdCBE as ptpTALECD. A study was conducted to identify favored positions of substituted cytosines by ptpTALECD (Nakazato et al., 2023). In this study, ptpTALECD_v2, which employed a modified version of DddA (DddA11’), could homoplasmically edit cytosines on the 3’ side of G and C, some of which were not homoplasmically edited by DdCBEs including ptpTALECD (Nakazato et al., 2023). C-to-T base editing in the plastid genome of rice was successfully achieved by introducing stop codons into the *psaA* gene of photosystem I, resulting in non-photosynthetic mutants that were unable to produce seeds (Li et al., 2021) (**Figure 7C**). Another group used a similar strategy to introduce a premature stop codon into the rice *psaA* gene, achieving near-homoplasmy (Zhang et al., 2024). A variant with high base-editing activity (DddA11), developed through an *in vitro* evolution system (Mok et al., 2022a), was adopted instead of DddA. Wang et al., 2024 developed a monomeric C-to-T base editor for rice plastid DNA. This base editor contains CTP, TALE, a cytidine deaminase for single-stranded DNA (ssDNA) [hA3A-Y130F (Ren et al., 2021b) or T_AD_C (Lam et al., 2023)], catalytically-inactivated DddA (DddA_E1347A_), and UGI in a tandem array. According to Cho et al (Cho et al., 2022), DddA_E1347A_ likely unwinds dsDNA, enabling the deamination of target Cs by the cytidine deaminase for ssDNA. This monomeric base editor targeted multiple Cs within and adjacent to the TALE-binding sequence with frequencies of up to 81%. Both groups integrated the transgene of the base editors into the rice nucleus via *Agrobacterium*-mediated transformation (Li et al., 2021; Zhang et al., 2024; Wang et al., 2024). Additionally, a transiently expressed TALE-FokI nickase, a cytidine deaminase for ssDNA, and an exonuclease, each fused with CTP, were employed to preferentially edit Cs on one of two DNA strands in plastids of rice protoplasts, with frequencies of up to 1.67% (Hu et al., 2024). The dimeric TALE-FokI nickase introduced a nick in between the TALE binding sequences, and the exonuclease subsequently removed nucleotides around the nick, generating ssDNA, which was then deaminated by the ssDNA-specific cytidine deaminase.

A-to-G base editors are now available for plastid DNA in *A. thaliana* (Mok et al., 2022b; Zhou et al., 2024; Mok et al., 2024), lettuce (Mok et al., 2022b), and rice (Zhang and Boch, 2023; Wang et al., 2024). These editors are based on those used for human mitochondrial DNA A-to-G base editing (Cho et al., 2022). One molecule of the dimeric A-to-G base editors named TALED (Mok et al., 2022b) and pTABE_v6 (Zhang and Boch, 2023) contains CTP, TALE, and the C-terminal half of DddA or its variant (DddA6). The other molecule contains CTP, TALE, the N-terminal half of DddA or DddA6, and an adenine deaminase for ssDNA (TadA8e). Specific adenines in between TALE-binding sequences were edited (nearly) homoplasmically in *A. thaliana* (Mok et al., 2022b) and rice (Zhang and Boch, 2023). These homoplasmic mutations in *A. thaliana* were inherited by the next generation (T_2_ plants). In addition, some of the T_2_ plants did not carry the nuclear transgene (so-called null segregants) and had no off-target mutations (Mok et al., 2022b). Transiently expressed TALED edited its target adenines in lettuce protoplasts with frequencies of up to 46% (Mok et al., 2022b). Mok et al. (2024) developed UDG-TALED by fusing uracil DNA glycosylase (UDG) to DddA, which removes uracil, a product of C-to-T editing. The UDG-TALED system showed similar efficiency to TALED in editing A:T pairs in lettuce and *A. thaliana*, but with significantly reduced C-to-T edits (Mok et al., 2024). Monomeric A-to-G base editors named mTALEAD (Zhou et al., 2024) or mTCABE-T (Wang et al., 2024) employed CTP, TALE, TadA8e (in mTALEAD) or a TadA variant T_AD_AC (in mTCABE-T), and DddA_E1347A_. Similarly to TALED, mTALEAD homoplasmically edited adenines around the TALE-binding sequence, and these homoplasmic base-edits were inherited by null-segregant T_2_ plants, which did not have off-target mutations (Zhou et al., 2024). The editor mTCABE targets both multiple cytosines and adenines around the TALE-binding sequence in rice with frequencies of up to 86% and 61.8%, respectively (Wang et al., 2024), due to T_AD_AC’s ability to deaminate both cytosine and adenine (Lam et al., 2023). Plastid genome base editing recently conferred resistance to the herbicides atrazine and metribuzin in *A. thaliana* (Mok et al., 2024; Nakazato et al., 2024). Atrazine resistance was achieved by introducing the S264G mutation in the D1 protein using UDG-TALED, while metribuzin resistance was achieved with V219I and A251V mutations using ptpTALECD and ptpTALECD_v2. In many species, the plastid genome is maternally inherited, so herbicide-resistant mutations in the maternal line’s plastid genome are passed on to all progenies (Chung et al., 2023).

Plastid genome-targeted base editing offers a better understanding of organellar genes necessary for respiration and photosynthesis and their role in enhancing desirable traits. Additionally, in many countries, null-segregant base-edited plants can bypass strict regulations required for cultivating genetically modified crops outdoors.

### 3.2 CRISPR/Cas9-mediated editing in chloroplasts

**Figure 8:**
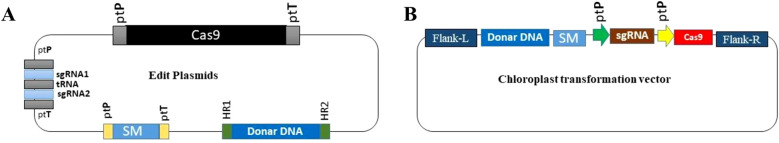
Schematic representation of the plasmid model constructs for CRISPR/Cas9 editing in chloroplasts. **(A)** Structures of Edit Plasmids used in editing *Chlamydomonas* chloroplasts (Yoo et al., 2020), (ptP-Plastid promoter, SM-selectable marker gene, HR1 and HR2-short homologous regions of wild plastid genome). **(B)** Map of chloroplast transformation vector used for tobacco chloroplast genome editing (Tang et al., 2020). ptP, plastid gene promoter; ptT, plastid gene terminator; SM, selectable marker gene; HR1 and HR 2, homologous regions 1 and 2; Flank-L, left flanking sequence; Flank-R, right flanking sequence.

Editing the chloroplast genome has remarkable potential to enhance crop modification, which in turn can help to meet the growing demands for agricultural sustainability and other future challenges. Interestingly, the RNA-guided genome editing tool CRISPR/Cas9 has been rapidly developed and widely used in plant nuclear gene editing (Li et al., 2013; Nekrasov et al., 2013). However, the application of CRISPR/Cas9-mediated editing in chloroplasts has been rarely reported, primarily due to the difficulty of simultaneously transporting and expressing both the guide RNA and cas9 protein into chloroplasts (Kim et al., 2021). Nevertheless, a few successful attempts have been made in CRISPR/Cas9-mediated chloroplast genome editing by overcoming the key obstacles to initiate the advanced chloroplast genome editing approaches. A new chloroplast genome editing technique that introduces “Edit Plasmids” in *Chlamydomonas reinhardtii* was successfully demonstrated. This method enabled induced donor DNA insertion at the target sites cleaved by Cas9/gRNAs in algal chloroplasts (Yoo et al., 2020) (**Figure 8A**). The INDEL mutations and substitutions at the target cleavage site with the “Edit Plasmids” approach were observed at a lower frequency. This suggests that homology-directed DNA repair (HDR) and replacement are the main outcomes of Cas9-induced cleavages in organelles. The Edit Plasmid can autonomously replicate and persist for an extended period in organelles to induce gene editing. Analysis of independent transgenic events for donor DNA insertion into Cas9/gRNA-driven target sites revealed an overall frequency of two positive events out of 20 independent transformants (10%). The construct employed in that study contained two guide RNAs (sgRNA1c and sgRNA2c) and Cas9 expressed under the strong *psaA* promoter. In another experiment, on-target mutations at one Cas9/gRNA target site were analyzed in chloroplasts transformed with Edit Plasmids containing or lacking the guide RNA (sgRNA2c) or donor DNA carrying Cas9 with different promoters (*psaA, psbD*, or none). SNPs resulting from Edit Plasmids (YP11 and YP31), which carry active Cas9 under the strong *psaA* promoter, led to the loss of an *AvaII* site in the chloroplast genome at a rate of 6.06%. Analysis of negative control Edit Plasmids without Cas9 (YP5) or guide RNA (YP29) showed a frequency of 7% for the loss of the *AvaII* site. Tang et al. successfully conducted a study involving the targeted delivery of CRISPR/Cas9 editing tools to tobacco chloroplasts using biolistics (Tang et al., 2020). The authors used editing tools to cut the specific target region between two homologous recombinant fragments to promote the integration of exogenous donor DNA. The efficiency of chloroplast transformation using this method was shown to increase six to ten-fold, demonstrating the potential to extend this technology to several other crop species (**Figure 8B**). However, future challenges include improving editing efficiency in different crop species, removing unmodified organellar genomes, and achieving complete homoplasmy.

### 3.3 Plastid-mediated RNA interference

**Figure 9:**
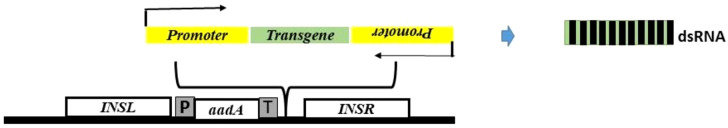
Schematic design of plastid transformation vectors for dsRNA expression. The targeted plastid genome regions with insertional sequence left (INSL) and insertional sequence right (INSR) are represented for recombination events. The strategy for cassette design to produce long dsRNA with specific promoter regions on either side of transgene, one with reverse orientation. The selectable marker gene *aadA* is driven by the promoter (P) with the 3’ untranslated terminator region (T).

Plastid-mediated RNA interference (PM-RNAi) has emerged as a viable alternative strategy to chemical pesticides for controlling invasive pests in several crop species (**Table 4**). This is achieved through a core mechanism that involves the uptake of dsRNA with a sequence that matches essential insect genes into the midgut cells of the insect and leads to disruption in the expression of the targeted gene(s) (**Figure 9**). Stable dsRNA expression was first studied in the plastids of *Nicotiana tabacum* and *Solanum tuberosum* with three different dsRNA constructs to target the Colorado potato beetle (CPB), one of the most invasive insect pests of solanaceous crops (Zhang et al., 2015a). The accumulation levels of ACT (encoding β-actin) dsRNA were found to be ~0.4% of the total cellular RNA, which is higher than those of SHR (encoding Shrub) and ACT+SHR dsRNA. The potential effects of CPB β-actin on three other potato pests have been evaluated in another study. Growth retardation and suppression of the *HvACT* (*Henosepilachna vigintioctopunctata* actin gene) gene was observed, with 91.7% identity to ACT of CPB (Ren et al., 2021a). A different approach, “RNAi-of-RNAi”, (improving the effect of PM-RNAi in transplastomic plants by treating with *in vitro*-synthesized fusion dsRNA) provides conceptual proof for enhanced insect resistance in potatoes. The authors generated transplastomic potato lines expressing dsRNA corresponding to *Shibire* (ds*SHI*) (encoding a dynamin protein) of CPB, which showed a less potent RNAi response than CPB *Shrub* (*St*-ds*SHR*) (encoding Notch receptor) or *β-actin* (*St*-ds*ACT).* However, an improved effect of PM-RNAi by *dsSHI* was achieved with a *dsRNase*-silencing approach, fusing dsRNAs targeting the *dsRNase1/2* genes. This significantly reduced CPB larva weight, with ds*SHI* accumulation levels up to 0.1% of the total cellular RNA (Li et al., 2023). The silencing of *cytochrome p450 monooxygenase* (P450)*, V* ***‐*** *ATPase*, and *Chitin Synthase (Chi)* genes via plastid-mediated RNAi in tobacco has been demonstrated. The disruption of target genes in the insect midgut led to stunted larval development and poor pupation in *Helicoverpa armigera.* The transcript abundance was found to be 3.5 million dsRNA copies per µg of total RNA (Jin et al., 2015). RNAi efficiency was assessed in another study by feeding *in vitro*-synthesized dsRNA of the 2222 bp *v-ATPase A* gene directly to larvae of *M. sexta*, inducing effective lethality via gene silencing. Whereas plastid transgene-derived dsRNA had not significant effect on survival, this suggests that the length of long dsRNA influences its low accumulation levels in plastids, which resulted in unproductive RNAi in *M.sexta* as compared to highly accumulated control short dsRNA (Burke et al., 2019). The control of *Frankliniella occidentalis* (Western Flower Thrip or WFT) by PM-RNAi was evaluated and published recently (Wu et al., 2022). Transplastomic tobacco plants expressing dsRNA and hairpin RNA (hpRNA) were generated to target four essential WFT genes: *ACT, TUB, VAT*, and *SNF* (**Table 4**). Despite using the same *Prrn* promotor, there was a considerable variation in RNA accumulation levels for both dsRNA and hpRNA. However, the results indicated that both acted as effective elicitors of RNAi responses and achieved better results than nuclear transgenic plants against WFT (Wu et al., 2022). An attempt to control sap-sucking insect pests by PM-RNAi was also established. The proportion of aphids feeding on transplastomic plants was lower, and these plants grew significantly faster with superior protection compared to nuclear transgenic plants (Dong et al., 2022). The PM-RNAi strategy was also validated in tomato plants expressing dsRNA of the *v-ATPase A* gene to control leaf-chewing and lacerate-and-flush feeding insects (Kaplanoglu et al., 2022). With the progression of research in plastid-mediated RNAi in a few crop species, there is a need to further expand the study range for economically important crops.

**Table 4:** Transplastomic plants expressing dsRNAs for crop pest control.

Crop plant	Targeted insect	Gene*	dsRNA accumulation levels (%)	References
Potato	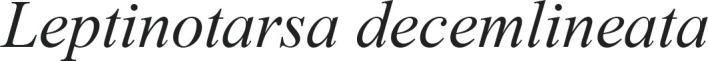	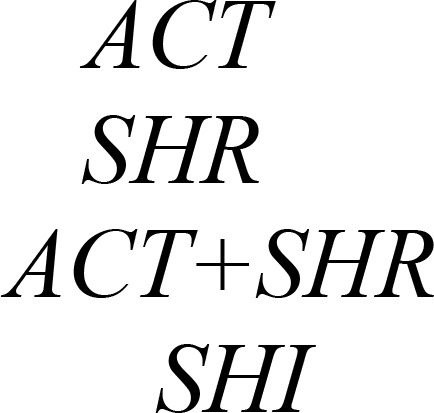	0.4%	Zhang et al., 2015a
			0.05%	
			0.1%	
			0.1%	Li et al., 2023
	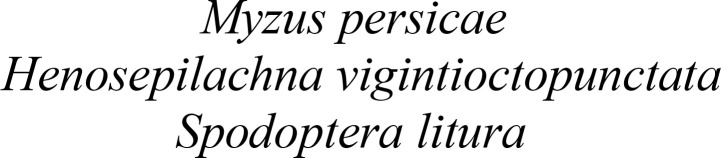	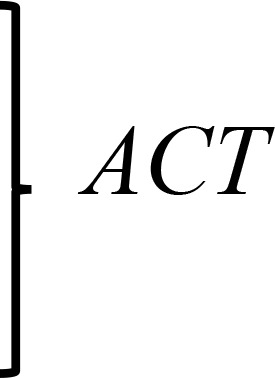	NI	Ren et al., 2021a
			NI	
			NI	
Tobacco	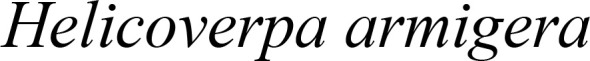	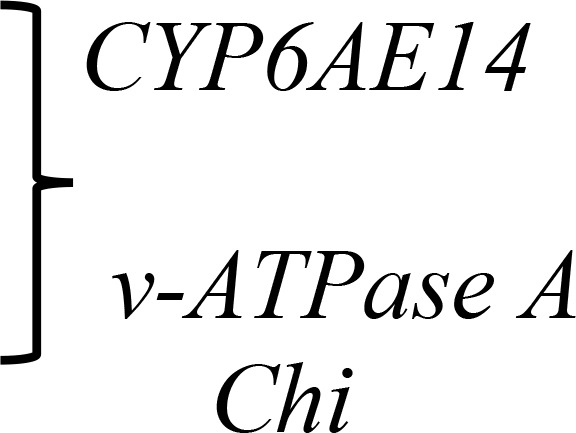	3.45 x10^7^	
			/µg total RNA	
			NI	Jin et al., 2015
			NI	
	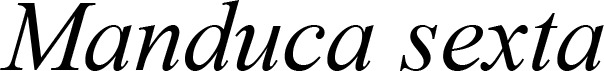	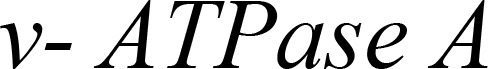	2.39x10^7^	Burke et al., 2019
			/500ng Total RNA	
	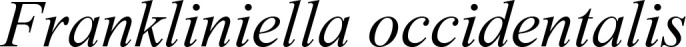	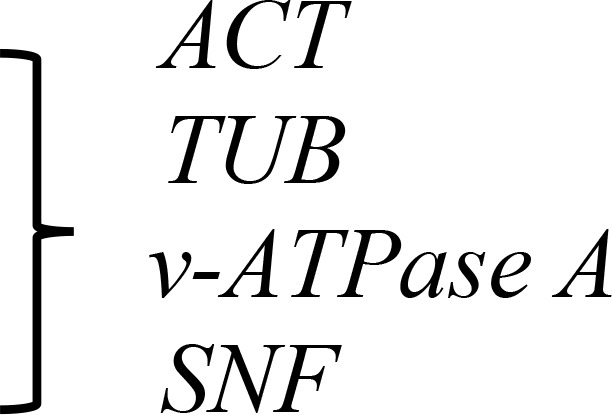	1%	
			0.8%	
			0.6%	Wu et al., 2022
			0.4%	
	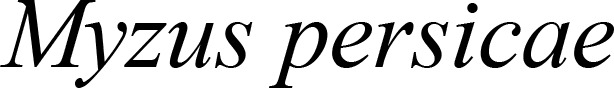	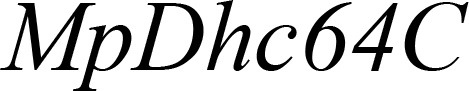	NI	Dong et al., 2022
Tomato	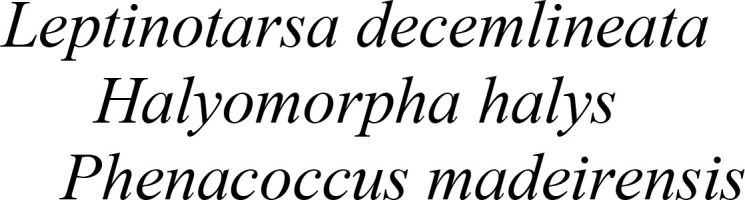	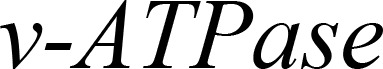	9.67x 10^7^	Kaplanoglu et al., 2022
			/500ng Total RNA	
	

NI: Not Indicated.

* *ACT*, β-actin; *SHT or also known as Vps32/Snf7*, Shrub; *SHI*, Shibire; *CYP6AE14*, Cytochrome *P450* monooxygenase; *v-ATPase A*, Vacuolar-type ATPase; *Chi*, Chitin synthase B; *TUB*, Tubulin; *SNF*, Transport III subunit SNF7; *MpDhc64C*, Heavy chain of dynein.

### 3.4 Metabolic engineering through plastid transformation

Synthetic biology approaches in chloroplast transformation allow for achieving desired expression levels by choosing appropriate combinations of plastid gene expression elements (e.g., promoters, Shine–Dalgarno sequences, and 5’ and 3’ untranslated regions). However, this requires several trial-and-error steps to optimize the expression constructs (Herz et al., 2005). Certain factors, such as the strength of different gene expression signals and the sequence of the coding region, can influence the level of foreign protein accumulation in chloroplasts, which subsequently can be unpredictable. Recent applications of synthetic biology methods have striven to advance and extend the toolbox to incorporate different metabolic pathways (Birch-Machin et al., 2004) (**Table 5**). One of the best-demonstrated examples is the introduction of a complex metabolic pathway for the synthesis of biodegradable plastic, polyhydroxybutyrate (PHB). In this study, transplastomic plants accumulated significant amounts of transgene-derived protein, showed severe growth retardation, and were infertile. Application and improvements of components for inducible expression of plastid transgenes could help achieve routine high-level protein production for the development of plants as biofactories for the economic production of biopolymers (Lössl et al., 2005).

**Table 5:** Applications of synthetic biology in implementing metabolic engineering in crop plants via plastid transformation.

Metabolic pathway	Crop plant	Target sites	Genes expressed *	References
Polyhydroxybutyrate (PHB) pathway	Tobacco	*trnN – trnR*	*phbA*, *phbB* & *phbC*	Lössl et al., 2005
Astaxanthin	Lettuce	*rbcL – accD*	*CrtW, CrtZ* & *Idi*	Harada et al., 2014
	Tobacco	*rbcL – accD*	*CrtW* & *CrtZ*	Hasunuma et al., 2008
Vitamin E	Tobacco & Tomato	*trnfM – trnG*	*HPT, TCY, TMT*	Lu et al., 2013
	Tobacco & Lettuce	*rbcL – accD*	*TC, ϒ-TMT* & TC+ϒ-TMT	Yabuta et al., 2013
Artemisinin production	Tobacco	*trnfM – trnG*	*FPS, ADS, CYP CPR* in plastids *CYB5, ADH1, ALDH1, DBR2&DXR* in nucleus	Fuentes et al., 2016
Dhurrin pathway	Tobacco	*trnfM – trnG*	*CYP79A1*, *CYP71E1* & *UGT85B1*	Gnanasekaran et al., 2016
β-carotene/provitamin A	Tomato	*trnfM – trnG*	*PSP*, *LCP LPP* & *LCE* *crtY* & *Lyc*	Wurbs et al., 2007; Apel and Bock, 2009
Luciferase pathway	Tobacco	*rps12 – TrnV* *trnI – trnA*	*luxCDABEG*	Krichevsky et al., 2010
Mevalonate pathway	Tobacco	*trnI – trnA*	*PMK, MVK, MDD, AACT, HMGS & HMGRt*	Kumar et al., 2012
Terpene pathway	Tobacco	*trnI – trnA*	FPS, SQS	Pasoreck et al., 2016

* *phbA*, β-ketothiolase; *phbB*, Acetoacetyl-CoA reductase; *phbC*, PHB synthase; *CrtW*, b-carotene ketolase; *CrtZ*, b-carotene hydroxylase; *Idi*, isopentenyl diphosphate isomerase; *HPT*, Phytyltransferase; *TCY*, Tocopherol cyclase; *TMT*, γ-tocopherol methyltransferase; *TC*, Tocopherol cyclase; *γ-TMT*, γ-tocopherol methyltransferase; *FPS*, Pyrophosphate synthase; *ADS*, Amorphadiene synthase; *CYP*, Cytochrome P450 monooxygenase CYP71AV1; CPR, Cytochrome P450 reductase; *CYB5*, Cytochrome b5 gene; *ADH1*, Alcohol dehydrogenase 1; *AlDH1*, Aldehyde dehydrogenase; *Dbr2*, Aldehyde delta-11(13) reductase; DXR, 1-deoxy-D-xylulose-5-phosphate reductoisomerase; *PSP*, Phytoene synthase; *LCP*, Lycopene b-cyclase; *LPP*, Lycopene b-cyclase/phytoene synthase fusion; *LCE*, lycopene b-cyclase; *crtY*, Lycopene b-cyclase; *Lyc*, Lycopene b-cyclase; *PMK*, Phosphomevalonate kinase; *MVK*, Mevalonate kinase; *MDD*, Mevalonate diphosphate decarboxylase; *AACT*, Acetoacetyl CoA thiolase; *HMGS*, HMGCoA synthase; *HMGRt*, C-terminal truncated HMGCoA reductase; *FPS*, Farnesyl diphosphate synthase; *SQS*, Squalene synthase .

The expression of a synthetic operon consisting of genes encoding b-carotene ketolase (*CrtW*) and b-carotenehydroxylase (*CrtZ*) from the marine bacterium *Brevundimona* ssp. strain SD212 in tobacco chloroplasts led to a 2.1-fold higher accumulation of isoprenoid units in transplastomic plants. This was indicated by a color change in the plants from green to reddish brown (Hasunuma et al., 2008). Similar studies have also been conducted in lettuce (Harada et al., 2014). An efficient strategy has been described for enhancing the vitamin E (tocochromanol pathway) content in tomato and tobacco through stable plastid transformation (Lu et al., 2013). A series of synthetic operons were designed with homogentisate phytyltransferase (*HPT*), tocopherol cyclase (*TCY*), and γ-tocopherol methyltransferase (*TMT*) genes for tobacco and tomato plants, and the resulting transplastomic plants showed an overall ten-fold increase in total tocochromanol (antioxidant) accumulation in leaves. Expression of *TC, ϒ-TMT*, and *TC+ϒ-TMT* genes for vitamin-E synthesis has also been established in lettuce (Yabuta et al., 2013). Engineering of complex biochemical pathways for artemisinin biosynthesis was efficiently carried out to achieve high levels of metabolic production of the precursor compound, artemisinic acid, in tobacco. This was done using a co-transformation approach of both plastome and nuclear genomes, termed COSTREL (Combinatorial Supertransformation of Transplastomic Recipient Lines). Results from this promising strategy revealed a 77-fold increase in the accumulation of artemisinic acid and also identified the key contributor for higher product yield, i.e., aldehyde dehydrogenase (ALDH1) (Fuentes et al., 2016). A pathway from sorghum was engineered into tobacco chloroplasts for the production of dhurrin, a cyanogenic glucoside that acts as a defense metabolite (Gnanasekaran et al., 2016). Metabolic engineering in tomatoes was carried out by expressing carotenoid biosynthesis genes and analyzing the provitamin-A content, which increased four-fold (Wurbs et al., 2007). Similar studies were conducted for the expression of lycopene β-cyclase from daffodil, which resulted in a 50% increase in carotenoid accumulation in tomato fruits (Apel and Bock, 2009). A complex metabolic pathway for the expression of the lux operon with six genes was accomplished, resulting in plants capable of emitting light visible to the naked eye (Krichevsky et al., 2010). Accumulation of higher levels of mevalonate, carotenoids, squalene, sterols, and triacylglycerols was observed in transplastomic plants by inserting the entire cytosolic MEV (Mevalonate) pathway (Kumar et al., 2012). The metabolic engineering pathway for squalene biosynthesis using farnesyl diphosphate synthase (*FPPS*) and squalene synthase (*SQS*) was achieved in tobacco (Pasoreck et al., 2016). Despite several benefits, plastid-mediated metabolic engineering has certain drawbacks, including potential interference with plastid architecture, development, and metabolism due to foreign metabolites. The generation of microcompartments may also be relevant in the context of plastid metabolic engineering by protecting the plastid from toxic metabolite products. In one study, morphologically accurate carboxysomes were produced by transforming a full set of carboxysome genetic components derived from the *H.neapolitanus* (halophilic archaea) genome into the tobacco chloroplast or plastid genome (Chen et al., 2023).

## 4 Challenges to overcome in plastid transformation technology

### 4.1 Limited range of target plant species

Although plastid transformation has been achieved in several dicotyledonous crop plants, similar successes have not yet been achieved in monocotyledonous plants, except for rice (Lee et al., 2006; Wang et al., 2018). This may be due to the lack of efficient *in vitro* plant regeneration protocols among these species and their resistance to the selection agents used in chloroplast transformation (Wang et al., 2018). Another major limitation is the poor expression of transgenes in non-green plastids (Zhang et al., 2012). For instance, the expression of the fused *p24-Nef* operon, driven by P*rrn*, was shown to generate 2.5% of total soluble protein (TSP) in green tomatoes but was not observed in ripened fruit. Genome-wide analysis of green tissues of tomato fruits and potato tubers has shown that two essential genes, *accD* (involved in lipid metabolism) and *clpP* (involved in proteolytic machinery), are thought to play a key role in non-green plastids. Therefore, promoters and 5′ UTRs associated with these genes may help with high transgene accumulation in non-green plastids (Kahlau et al., 2006; Valkov et al., 2009).

### 4.2 Pleiotropic effects in transplastomic plants

In most reported studies, recombinant protein expression in plastids does not impact the progression of plant growth and development. However, some researchers have reported irregularities in phenotypic characteristics, such as yellowing of leaves, reduced plant growth, and infertility in transformed plants linked to expression of certain plastid transgene-derived proteins. Slower growth and a chlorotic appearance were observed, along with a reduction in protein levels by up to 50% when the HIV-1 Pr55^gag^ polyprotein was produced in tobacco chloroplasts (Scotti et al., 2009). Similarly, 30% of transplastomic lines exhibited yellow tissues with the expression of HIV-1 protein^Nef^ in tobacco and tomato plants (Zhou et al., 2008). The expression of the A27L immunogenic antigen from the vaccinia virus in transplastomic lines did not cause any changes in flowering or seed production, but resulted in reduced growth and a pale green appearance (Rigano et al., 2009). To date, the reasons behind these effects and their solution remain unclear, but a few clues have emerged to explain the connection between an abnormal physical appearance and the specific gene being activated (Ruiz and Daniell, 2005). Excessive production of foreign proteins may have negative impacts, such as disrupting the structure and function of thylakoids, activating new reading frames involved in cytoplasmic metabolism, or reducing ATP production levels (Pelletier and Budar, 2007). Recently, the application of protein fusions, such as SUMO (small ubiquitin-like modifier) in the expression of multiple AMPs has led to increased protein accumulation and reduced phenotypic effects (Hoelscher et al., 2022).

The potential unintended side effects on native chloroplast gene expression due to transgene insertion and subsequent high-level expression through plastid transformation have been comprehensively studied. Chloroplast gene expression within the *petL-petG-psaJ-rpl33-rps18* transcription unit at three different transgene integration sites was analyzed by targeted ribosome and transcriptome profiling approaches (Ghandour et al., 2023). The data suggests that transcriptional read-through occurs frequently, with an overaccumulation of transcripts from downstream sense-oriented genes. However, there are reduced transcriptional and translational activities in downstream antisense-oriented native chloroplast genes. Together, the findings provide a deep understanding that can help in choosing insertion sites and other components for transgenic expression in plastids without influencing nearby native genes.

### 4.3 Lack of commercialization of plastid technology

Despite outstanding achievements at the laboratory scale, chloroplast transformation technology has not yet reached the commercial field level. Promising developments have emerged to streamline the transformation process by adopting advanced cloning methods and new selectable markers in the construction of expression vectors. The recent FDA (Food and Drug Administration) approval of IND (Investigational New Drug) CTB-ACE2 (cholera toxin B fused angiotensin-converting enzyme 2) chewing gum (Ganesan et al., 2023) paved the way for a Phase I/II placebo-controlled, randomized double blind clinical trial to advance CTB proinsulin to clinics and develop affordable oral insulin (Daniell et al., 2023). A few studies have demonstrated downstream technologies for protein purification using temporary immersion bioreactors (TIBs) for transgenic plant biomass propagation (Michoux et al., 2013). More recently, Wu et al. (2025) outlined the experimental procedures for designing transformation constructs for PEP purification from transplastomic tobacco plants (Wu et al., 2025). Synthetic elements are emerging as key tools for the advancement of chloroplast engineering and transformation methods, driving this technology towards field level applications (Scharff and Bock, 2014).

## 5 Concluding remarks

Chloroplast engineering provides a promising opportunity for expressing various industrial and agricultural traits to meet the growing food demand from an increasing world population. Advances in chloroplast transformation have demonstrated its potential in the fields of agriculture (Harada et al., 2014; Lin et al., 2014), phytoremediation (Ruiz et al., 2011), and biopharmaceuticals (Boyhan and Daniell, 2011). Progress in transplastomic technology has highlighted its importance as an ideal bio-factory for the production of antigenic vaccines (Su et al., 2015). Synthetic biology approaches are being explored for vector design, DNA delivery, and gene regulation to advance plastid engineering research. However, plastid transformation in non-green tissues remains elusive (Bock, 2015). Recent advancements in plastid engineering and transformation methods have created new opportunities for exploring precise genome-level editing. Despite numerous challenges, plastid transformation holds the potential to enable cost-effective production of recombinant proteins and eliminate socio-environmental issues associated with nuclear genome-modified crops.
